# Social Exclusion Changes Histone Modifications H3K4me3 and H3K27ac in Liver Tissue of Wild House Mice

**DOI:** 10.1371/journal.pone.0133988

**Published:** 2015-08-12

**Authors:** Linda Krause, Bernhard Haubold, Angelika G. Börsch-Haubold

**Affiliations:** 1 Institute of Computational Biology, Helmholtz Zentrum München, Neuherberg, Germany; 2 Department of Evolutionary Genetics, Max-Planck-Institute for Evolutionary Biology, Plön, Germany; University of Florida, UNITED STATES

## Abstract

Wild house mice form social hierarchies with aggressive males defending territories, in which females, young mice and submissive adult males share nests. In contrast, socially excluded males are barred from breeding groups, have numerous bite wounds and patches of thinning fur. Since their feeding times are often disrupted, we investigated whether social exclusion leads to changes in epigenetic marks of metabolic genes in liver tissue. We used chromatin immunoprecipitation and quantitative PCR to measure enrichment of two activating histone marks at 15 candidate loci. The epigenetic profiles of healthy males sampled from nest boxes differed significantly from the profiles of ostracized males caught outside of nests and showing bite wounds indicative of social exclusion. Enrichment of histone-3 lysine-4 trimethylation (H3K4me3) changed significantly at genes *Cyp4a14*, *Gapdh*, *Nr3c1*, *Pck1*, *Ppara*, and *Sqle*. Changes at histone-3 lysine-27 acetylation (H3K27ac) marks were detected at genes *Fasn*, *Nr3c1*, and *Plin5*. A principal components analysis separated the socialized from the ostracized mice. This was independent of body weight for the H3K4me3 mark, and partially dependent for H3K27ac. There was no separation, however, between healthy males that had been sampled from two different nests. A hierarchical cluster analysis also separated the two phenotypes, which was independent of body weight for both markers. Our study shows that a period of social exclusion during adult life leads to quantitative changes in histone modification patterns in mouse liver tissue. Similar epigenetic changes might occur during the development of stress-induced metabolic disorders in humans.

## Introduction

Laboratory-bred house mice that are released into a semi-natural enclosure quickly form a social structure [1,2]. Males initially explore the area and start fighting over territories. Once the hierarchy is established, dominant males keep patrolling their territory and fight intruders [2,3]. They start breeding with one or more females in their territory and family groups form in nest boxes. When the first generation born in the enclosure reaches sexual maturity, younger males are integrated as subordinate males and nest boxes are occupied with growing breeding units. Subordinate males tend to hide in bedding material, restrict their movements to short visits to nearby food and water, and retreat to their nests when they are attacked [3]. Depending on the experimental set-up and the group size, several types of dominant behavior have been observed. Poshivalov [4] describes two main types of male dominant behavior. In groups of four, the dominant male unilaterally attacks the subordinates, but there are no fights between the subordinates (despotic dominance). In groups of twelve, he observed partial hierarchy, in which subordinates also attack other subordinates. The dominant male has a strong rival that he attacks more often than other subordinates. The rival responds with reciprocal attacks and may succeed in gaining dominance. Fighting relationships among male mice have further been classified as exclusive dominance by one male, dominance with various forms of resistance from sub-dominant males, a linear order of dominance with or without resistance, unsettled dominance, or no fighting [5].

Enclosure experiments running for a several months may lead to an extreme form of subordination, the socially excluded, or ostracized, males. In an experiment running over three months Crowcroft [6] found that males which were physically in poor condition shared a nest box. He judged these males to be of low social status because there were never any females in this nest. Reimer and Petras [2] observed an increase in fighting among males after 5.5 months, and after 8 months, 63% of males carried bite wounds. Over several weeks, twelve badly bitten males shared a nest box containing no females. In an experiment by Anderson and Hill [7] a quarter of the subordinate males were eventually killed whereas the others remained subordinate. Crowcroft and Rowe [8] recorded that older males became the object of aggression in crowded pens, which lead to bite wounds in 15% to 56% of the males, and in extreme cases to the denudation of fur. Females were much less affected by bites [2,7,8].

We have observed the development of similar social structures in house mouse populations living in enclosures [9,10]. Socially excluded males appeared at high population densities: they did not hide in nest boxes when a person entered the enclosure, and when trapped, they showed numerous bite marks and patches of thinning fur. These animals were regularly removed from the enclosures.

Many examples of how social stress influences transcript levels and DNA methylation in gene promoter regions come from experiments that investigate the role of early life stress on adult behavior [11–15]. For example, brief separations of rat pups from their nursing mothers causes an increase in glucocorticoid receptor (*Nr3c1*) transcript levels in the hippocampus and frontal cortex during adult life [16]. Similarly, offspring of female rats that display a high licking-grooming behavior have an increased hippocampal *Nr3c1* expression, which is caused by low levels of DNA methylation in the corresponding promoter region [17]. It has been proposed, that an increase in licking and grooming by the mother after the reunion with her pups mediates the response to separation by DNA promoter hypomethylation at the hippocampal glucocorticoid receptor [15]. A change in *Nr3c1* expression and promoter DNA methylation has also been described from post-mortem examination of human hippocampal tissue in victims of childhood abuse [18]. A similar methylation change was found in newborn umbilical cord blood after mothers experienced prenatal stress [19].

Apart from nutrition, toxins, and seasonal changes, the social environment is influencing epigenetic plasticity during adult life [20–22]. Social defeat is one of the most severe sources of stress since it entails continuous exposure to the stressor. Compared to other experimentally inflicted stressors such as forced swimming, electric foot-shocks, noise or cold, social defeat leads to the highest stress response in male rats as measured by plasma corticosterone and catecholamine levels [23]. The infliction of chronic social defeat induces depression in a mouse model, and the transcriptional downregulation of the gene *Bdnf* in the hippocampus is accompanied by an increase in the repressive histone mark H3 lysine-27 dimethylation [24]. It is well known that neuroendocrine changes induce physiological responses such as increases in blood pressure, heart rate, blood glucose and insulin, and biochemical responses such as a decrease in liver glycogen, elevated gluconeogenesis and lipolysis [25–27]. However, in contrast to studies on brain tissue, where transcript changes can be explained by epigenetic changes, the effect of stress on epigenetic settings in the organs affected by the brain's responses are less well researched.

We have previously measured epigenetic differences in wild mouse liver tissue as a result of an environmental change in diet and day-light [10]. We detected quantitative changes in activating histone marks, which were small but were concentrated in biochemical pathways that were concordant with the experimental set-up and thus presented a response to the metabolic challenge. The aim of the present study is to investigate whether the phenotype of ostracized males that we observe in our mouse enclosure populations had altered histone marks in comparison to healthy males caught in nest boxes as part of a family group. We call these two phenotypes "ostracized" and "socialized" males, respectively. We quantify two histone modifications that mark active genes, trimethylation at lysine 4 of histone 3 (H3K4me3) and acetylation at lysine 27 of histone 3 (H3K27ac) by chromatin immunoprecipitation (ChIP) and quantitative PCR (qPCR) within a region of the gene transcription start site (TSS). In accordance with known metabolic changes that might occur in stressful situations [25,27–29], we analyzed nine loci that are part of the peroxisome proliferator-activated receptor alpha (Ppara) signaling pathway, an important regulator of lipid metabolism in liver, and a few other genes that showed changes in previous experiments. We found significant differences of histone modifications at several loci, and both a principal components analysis (PCA) and a hierarchical clustering analysis separated the histone marks of ostracized and socialized males.

## Results

### Social hierarchies in the mouse population

At the time of sampling, the wild house mouse population consisted of 408 mice. We classified all mice by location (nest boxes: A—J; free-roaming: Z), body weight, sex, and fur condition (healthy or wounded; Table 1). We defined three age groups in accordance with typical body weights of cage-reared mice from our wild mouse population: pups weighing less than 8 g (up to 22 days old), prepubertal animals weighing 8–13 g (approximately up to 30 days old), and adult animals weighing more than 13 g. All ten nest boxes were occupied by family or breeding units with on average 10.6 ± 8.4 adult males and 9.9 ± 4.4 adult females (mean ± sd). Most of these animals were healthy and did not show any signs of social repression: In six nest boxes, there were no wounded males, in three houses, there was one, and in one house there were two (Table 1). This corresponded to a fraction of wounded males of 0.1–0.2 in houses D, E, and I. This fraction was higher in house J because there were only two adult males present with one male showing bite marks. Of the 58 free-roaming males, 22 were deemed ostracized by their number of bite marks. This corresponded to a fraction of 0.56. The fraction of wounded, free-roaming females was only 0.13 (Table 1). Young animals with bite wounds were rare (3 pups, 1 young male, not shown). These results are in agreement with previous descriptions of enclosure populations [2,8].

**Table 1:** Population structure at time point of sampling of healthy/socialized and wounded/ostracized males.

Nest boxes^a^	A	B	C	D	E	F	G	H	I	J	Z	Total
Number of mice	41	38	41	45	41	48	25	31	7	33	58	408
♂ > 13 g	22	1	14	10	25	2	12	13	5	2	39	145
♀ > 13 g	12	7	17	12	12	14	9	9	2	5	15	114
♂ 8–13 g	3	3	4	4	1	5	1	4	0	0	0	25
♀ 8–13 g	3	2	4	4	1	0	3	2	0	2	0	21
Pups < 8 g	1	25	2	15	2	27	0	3	0	24	4	103
Sex ratio^b^ > 13 g	0.65	0.13	0.45	0.46	0.68	0.13	0.57	0.59	0.71	0.29	0.72	0.56
Sex ratio^b^ 8–13 g	0.50	0.60	0.50	0.50	0.50	1.00	0.25	0.67	NA	0.00	NA	0.54
Weight ♂ > 13 g: mean	20.5	22.7	19.1	18.9	21.4	18.3	20.2	20.6	24.3	19.8	23.7	
Weight ♂ > 13 g: sd	4.1	NA	2.8	3.4	3.8	5.0	1.9	2.9	3.5	8.3	3.6	
Weight ♀ > 13 g: mean	22.8	23.1	20.9	22.8	22.5	27.5	19.2	22.6	34.3	23.0	24.3	
Weight ♀ > 13 g: sd	11.2	6.5	5.7	5.5	8.5	7.0	4.7	7.8	0.2	8.4	7.7	
Number of wounded ♂ > 13 g	0	0	0	1	2	0	0	0	1	1	22	27
Fraction wounded ♂ > 13 g	0	0	0	0.10	0.08	0	0	0	0.20	0.50	0.56	0.19
Number of wounded ♀ > 13 g	0	0	0	0	0	2	0	1	0	0	2	5
Fraction wounded ♀ > 13 g	0	0	0	0	0	0.14	0	0.11	0	0	0.13	0.04
Number of analyzed healthy ♂ > 20 g	8				4	1	4	8				25
Number of analyzed ostracized ♂ > 20 g											16	16

^a^A-J: nest boxes; Z: free-roaming mice at the time of monitoring.

^b^Sex ratio is the fraction of males.

For the analysis of epigenetic marks, we selected 25 healthy males from nest boxes A, E, F, G, H and 16 free-roaming, wounded males. The first group represented socialized males, the second group represented ostracized males. The group of socialized males weighed 22.7 ± 1.8 g, the group of ostracized males 25.4 ± 2.3 g (mean ± sd). This difference is significant as judged by a two-tailed Student’s t-test (p = 0.0002). The higher weight of ostracized males may indicate a larger fraction of older males in this group. S1 Table lists phenotypic data of all mice analyzed and shows the assignment of samples to two separate ChIP-DNA preparations. As a control, we also tested possible influences of body weight and sample preparation (see below).

### Histone modifications H3K4me3 and H3K27ac

We quantified DNA bound to two activating histone modifications by ChIP-qPCR from liver tissue of socialized and ostracized males. We selected 15 loci, twelve of which are associated with fat metabolism as cell surface receptors, nuclear factors (transcription factors) and metabolic enzymes, and with cholesterol metabolism (Table 2). The house-keeping gene *Gapdh* was included as a control. In addition, we measured two loci, *Igfbp2* and *Serpina6*, that showed environmentally-induced H3K4me3 changes in previous experiments [10]. The quality assessment of the primer pairs that we designed is described in the Methods Section.

**Table 2:** Names and function of 15 genes that were selected for epigenetic analysis.

Gene	Name or Specific Function	Functional Class	Literature
*Gapdh*	glyceraldehyde 3-phosphate dehydrogenase	house-keeping gene	[30]
*Cd36*	fatty acid translocase	receptor	[31,32]
*Slc27a5*	fatty acid transporter	receptor	[33]
*Ppara*	peroxisome proliferator activated receptor alpha	transcription factor	[32,34]
*Pparg*	peroxisome proliferator activated receptor gamma	transcription factor	[32,34]
*Acox2*	acyl-Coenzyme A oxidase 2, branched chain	target gene	[34]
*Cyp4a14*	cytochrome P450, family 4, subfamily a, polypeptide 14	target gene	[35]
*Fasn*	fatty acid synthase	target gene	[32,34]
*Nr3c1*	glucocorticoid receptor	target gene, transcription factor	[34]
*Pck1*	cytosolic phosphoenolpyruvate carboxykinase 1	target gene	[34]
*Insig2*	insulin induced gene 2	cholesterol synthesis regulator	[36]
*Plin5*	cellular neutral lipid accumulation	lipid storage/trafficking	[10]
*Igfbp2*	insulin-like growth factor binding protein 2	regulation of cell growth	[37]
*Sqle*	squalene epoxidase (cholesterol biosynthesis)	target gene	[32,38]
*Serpina6*	transcortin	sterol hormone carrier	[39]

**Fig 1:**
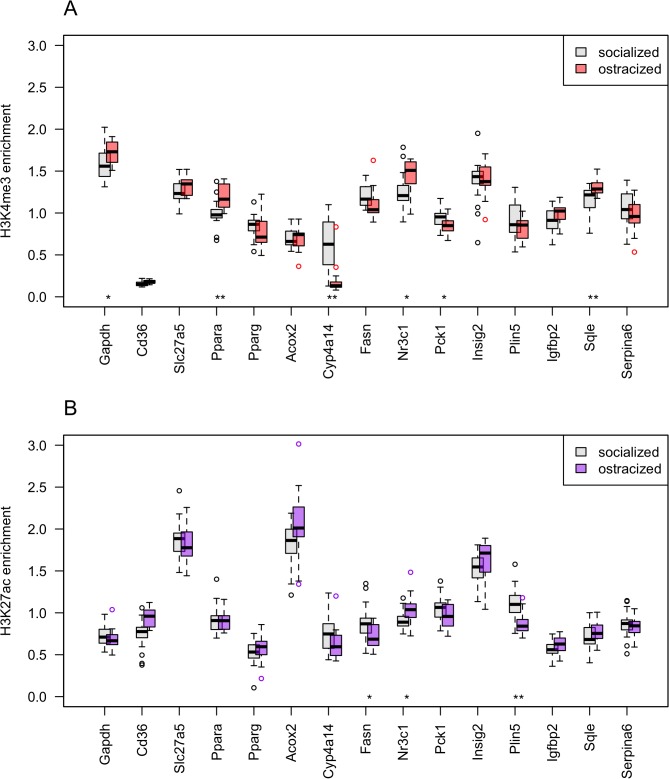
Enrichment of DNA in histone immunoprecipitates in relation to the input. (A) H3K4me3 enrichment was analyzed at 15 loci for 24 socialized males (housed and healthy) and 15 ostracized males (free roaming and wounded). (B) H3K27ac enrichment was analyzed for 25 socialized and 16 ostracized males. Significant differences in means were determined by a permutation test and corrected for multiple testing. Results are indicated by * for p ≤ 0.05 and by ** for p ≤ 0.01.

The histone immunoprecipitation resulted in varying enrichment of epigenetic marks at the selected loci in relation to the input control sample (Fig 1). In order to connect measurements and mice, we also labeled each data point with the mouse ID from S1 Table (S1 Fig). The mouse IDs show that the highest and lowest marking levels per locus did not consistently come from the same individuals. In contrast, consistently high or low outliers were observed when Gapdh was used for normalization. For example, the data from mice g1 and z16 were constantly at maximum or minimum outlier positions in the H3K4me3 dataset after Gapdh normalization (S1 Fig). The mean normalization, as shown in Fig 1, centers the dataset and gives for each mouse an individual pattern of histone modification enrichment over the selected 15 loci. However, it does not prevent the presence of outlier samples, because their data values switch between extreme low and high, i.e. produce a large standard deviation (see Methods).

In order to numerically compare enrichment results between the loci and between the two phenotypes, means and standard deviations of the data shown in Fig 1 are also given in S2 Table. In the H3K4me3 immunoprecipitates, strongest enrichment was measured for *Gapdh*, *Fasn*, *Nr3c1*, and *Insig2* (Fig 1A). In the H3K27ac precipitates, the strongest marks were at two different loci, at *Slc27a5* and *Acox2* (Fig 1B). *Insig2* was relatively strong for both markers. Variation between mice was largest at locus *Cyp4a14* from the H3K4me3 immunoprecipitates, where some mice seem to be missing the marking altogether. Variation at locus *Acox2* was much higher for H3K27ac than for H3K4me3. Thus, although both H3K4me3 and H3K27ac mark active TSS, the relative enrichment patterns were distinct at the 15 loci studied. Normalization by *Gapdh* in the enrichment calculation resulted in similar relative differences within both histone modification datasets (S1 Fig; S2 Table).

Whereas *Gapdh* was the strongest marker in the H3K4me3 immunoprecipitates, it was a relatively weak marker in the H3K27ac datasets (Fig 1). The opposite was observed at *Acox2*. This discrepancy was not due to the position of the qPCR amplicon with respect to peak positions of H3K4me3 and H3K27ac ChIP-Seq experiments. We had designed the positions of all qPCR amplicons with the help of our previously published H3K4me3 ChIP-Seq data from livers of wild mice [10] that we visualized as custom tracks on the UCSC mouse genome browser [40]. We also tested the amplicon positions against ChIP-Seq peaks from the H3K4me3 and H3K27ac adult mouse liver tracks of the Mouse ENCODE Consortium [41]. S2 Fig shows that the qPCR amplicons of *Gapdh* and *Acox2* were in the middle of ChIP-Seq peaks for the two histone markers investigated. The ChIP-Seq peaks at locus *Cyp4a14* from lab strain C57Bl/6 mice were low (S2 Fig) compared to the two other loci and compared to our wild mouse data. Our qPCR data for *Cyp4a14* showed low marks in some wild mice but also substantial marks in other individuals (Fig 1).

### Statistical analysis of individual loci

Statistical significance of differences in enrichment between socialized and ostracized mice was assessed by Monte Carlo tests. Significant differences of means between phenotypes were found at six loci for H3K4me3 and at three loci for H3K27ac (Table 3). At these loci, the direction of change was the same for both histone modifications. In the ostracized males, compared to the socialized males, upmarked genes were *Nr3c1*, and *Sqle*, and downmarked genes were *Cyp4a14*, *Fasn*, *Pck1*, and *Plin5* (S2 Table). Because the ostracized males were on average heavier than the socialized males (S1 Table), we also tested whether epigenetic marks differed between light and heavy males. When we compared the lightest 16 males to the heaviest 16 males, only the H3K27ac mark at *Plin5* was significantly different (Table 3). The changes in H3K27ac marks at *Plin5* that we observed between the two phenotypic groups may thus have a component related to body weight. Similar results were obtained by conventional statistical tests (S3 Table). Variances did not differ significantly between the two phenotypic groups or between the two weight groups (S3 Table).

**Table 3:** P-values of testing H3K4me and H3K27ac enrichment data for equality of means by permutation.

Marker	H3K4me3		H3K27ac	
Gene	Phenotype	Weight	Phenotype	Weight
*Gapdh*	0.050	0.406	0.453	0.345
*Cd36* ^a^	0.029	0.802	0.002	0.429
*Slc27a5*	0.150	0.140	0.453	0.353
*Ppara*	0.001	0.986	0.967	0.315
*Pparg*	0.259	0.986	0.368	0.815
*Acox2*	0.883	0.986	0.155	0.788
*Cyp4a14*	4 x 10^−4^	0.349	0.155	0.315
*Fasn*	0.129	0.986	0.025	0.971
*Nr3c1*	0.015	0.986	0.018	0.407
*Pck1*	0.050	0.986	0.092	0.315
*Insig2*	0.883	0.986	0.301	0.815
*Plin5*	0.168	0.986	6 x 10^−5^	0.002
*Igfbp2*	0.119	0.986	0.262	0.815
*Sqle*	0.009	0.986	0.344	0.755
*Serpina6*	0.259	0.986	0.453	0.501

Phenotype comparisons were carried out between 24 socialized and 15 ostracized males (H3K4me3) and between 25 socialized and 16 ostracized males (H3K27ac). Weight comparisons were carried out between 16 light and 16 heavy males. Permutation tests for equality of means were performed with 10,000,000 iterations per run; given is the mean P-value from 3 runs. P-values were adjusted using the Benjamini-Hochberg method.

^a^Since the enrichment at locus *Cd36* was < 2-fold over negative control, results for this locus need to be treated with caution.

### Statistical analysis of histone enrichment datasets by Manova

A Manova analysis was performed on the mean-normalized data of 14 loci (see Methods) to test whether the socialized and ostracized mice were separated by histone modifications. The H3K4me3 marker separated the two phenotypes with a P-value of 9.9 x 10^−5^. The inclusion of the two samples that had shown untypically high variance in the H3K4me3 raw data and that had been excluded from statistical analyses (see Methods) did not change this result (p = 2.4 x 10^−4^). The H3K27ac marker also significantly separated the two phenotypes (p = 3.1 x 10^−6^).

We used the Manova method to explore other possible group classifications, such as comparing two weight groups, as well as two groups of socialized males. In addition, we controlled for the two independent sample preparations. Separating mice by body weight in two groups of 16 individuals each did not result in a significant difference of histone modifications, and neither did the comparison of the lightest with the heaviest mice (n = 12–14, S4 Table). The comparison of eight socialized mice from one nest with eight socialized mice from another nest did not result in splitting these two groups; neither did the sample preparation controls (S4 Table). These results indicate that the histone modification differences between socialized and ostracized males were not caused by a difference in body weight, or indirectly, in age.

### PCA

**Fig 2:**
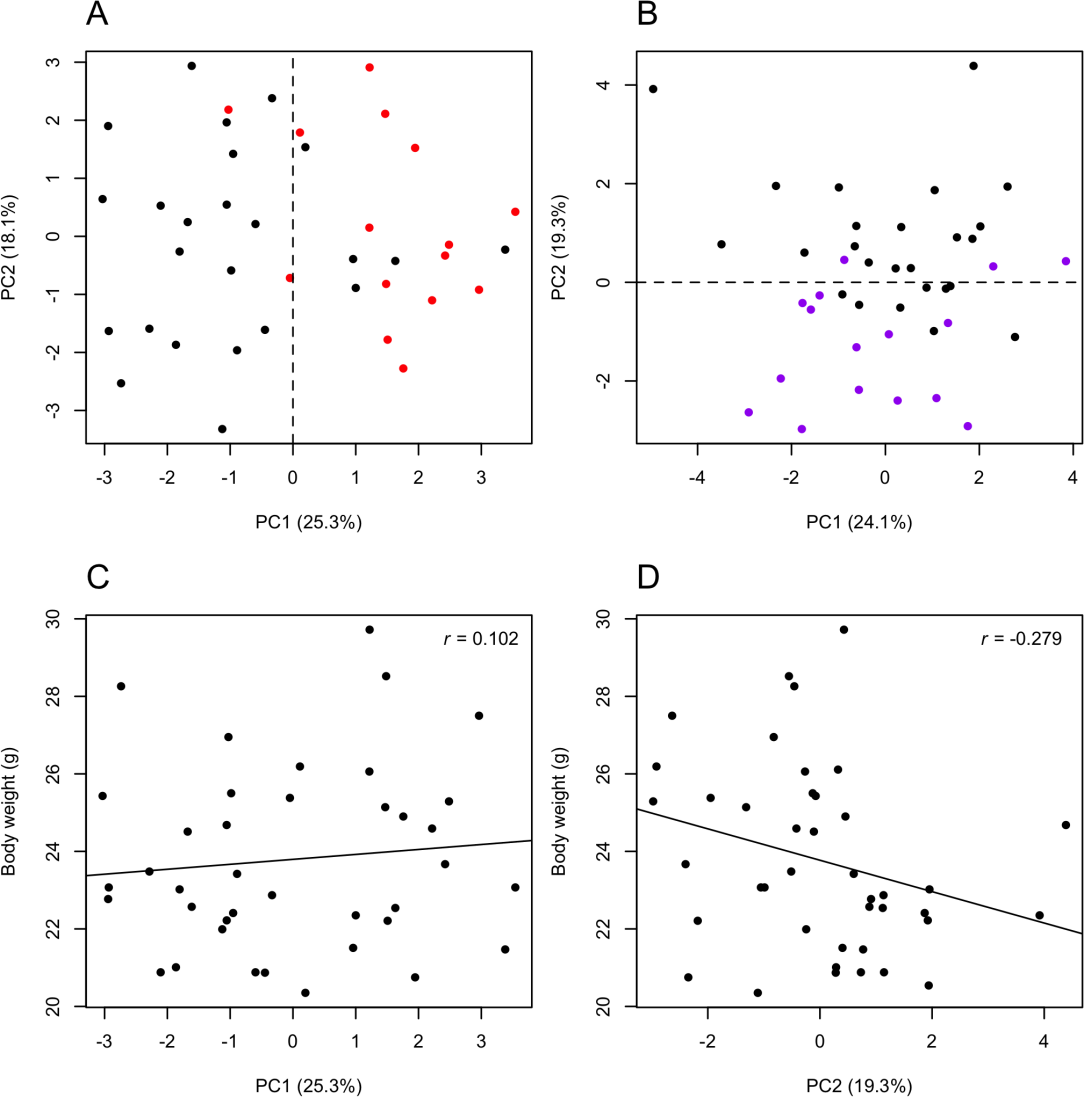
PCA of H3K4me3 and H3K27ac enrichment data. The percentage of the PC axis label is the fraction of variance that is explained by the PC. (A) Separation of the H3K4me3 enrichment data from 39 mice and 14 loci into two phenotypes: black are the socialized (n = 24) and red are the ostracized (n = 15) males. (B) Separation of the H3K27ac enrichment data from 41 mice and 14 loci into two phenotypes: black are the socialized (n = 25) and purple are the ostracized (n = 16) males. The dashed lines indicate the split into two groups that is used to test significance by permutation. (C) Correlation of PC1 prediction values from H3K4me3 enrichment and body weight. (D) Correlation of PC2 prediction values from H3K27ac enrichment and body weight. The black lines are linear regression lines and r is the Pearson's product-moment correlation; neither correlation was significant.

We tested whether PCA separated the histone mark enrichment data into the two phenotypes and the significance of separation was assessed by permutation. For the H3K4me3 mark, the separation at the level of PC1 = 0 was highly significant (p = 7.6x10^-5^, Fig 2A), but not at lower PCs (see PC1 to PC5 in S3 Fig). For the H3K27ac mark, the separation along PC2 = 0 was significant (p = 3.6x10^-3^, Fig 2B). Again, lower PCs did not separate the two phenotypes (shown up to PC5 in S4 Fig). The proportion that a PC contributed to the overall variance was similar between the two epigenetic markers. For example, PC1 explained 25% and 24% of the variances, respectively, PC2 18% and 19% (Fig 2A and 2B), and PC5 9% and 8% (S3 Fig, S4 Fig). To test whether or not body weight influenced the PCA separation, we correlated the H3K4me3 PC1 values with body weights (Fig 2C) as well as the H3K27ac PC2 values with body weight (Fig 2D). Both correlations were not significant as assessed by the Pearson's product-moment correlation (H3K4me3: p = 0.5; H3K27ac: p = 0.077). However, Spearman's rank correlation (rho = -0.36) showed marginal significance with a P-value of 0.02 for the H3K27ac dataset.

To visualize the contribution of body weight in the PCAs, we divided the mice into three weight groups and colored the PCA plots in black for light mice, grey for medium mice, and green for heavy mice. There was no separation by body weight for marker H3K4me3 up to PC5 (p = 0.7, S5 Fig). However, for marker H3K27ac PC2 separated two weight groups light (20.0–22.4 g, n = 14) and heavy (25.0–30.0 g, n = 13) with a P-value of 6.3x10^-3^ (group split at PC2 = 0, S6 Fig). There was also a significant separation by PC4 (group split at PC4 = 0, p = 0.0014, S6 Fig), which we did not observe in the phenotype annotation (p = 0.7, S4 Fig). It is possible that the strong difference at locus *Plin5* in the H3K27ac enrichment data, as shown in Fig 1B and Table 3, influenced the body weight correlation for this epigenetic marker. The PCA shows that the changes in the H3K4me3 modification that grouped socialized and ostracized mice were independent of body weight, whereas there was a partial dependence for the H3K27ac modification.

Gapdh-normalized datasets gave qualitatively similar results. H3K4me3 enrichment data separated the two phenotypes along PC1 and PC2 (p = 3.1x10^-3^, S7 Fig). With 35.5%, PC1 contributed to a larger fraction of the variance of the dataset than PC1 of the mean-normalized data (25.3%, Fig 2A). For the H3K27ac modification, PC2 and PC3 separated the data into the two phenotypes (p = 1.1x10^-5^, S8 Fig). The contributions of PC2 and PC3 to data variance were smaller than in the mean-normalized dataset (S4 Fig).

We also used the PCA analysis to test for separation of healthy males between different nest boxes and for laboratory procedures. The two phenotypic groups did not separate in a comparison of eight males from nest A with eight males from nest H (S9 Fig). Similarly, the independent ChIP preparations by two experimentalists were not visibly grouped by PCA (S10 Fig). There was a possible separation at PC2 = 0 in the H3K4me3 dataset but this was not significant (p = 0.055). The level of this separation did also not correspond to the separation of the two phenotypes as described above and shown in Fig 2A. These results are in agreement with the Manova tests where eight males from two houses and comparisons of two chromatin preparations did not give significant results (S4 Table).

### Hierarchical cluster analysis

In addition to statistical tests and PCA, we subjected the histone modification data to hierarchical cluster analysis in order to test whether pairwise distances separated the socialized from the ostracized mice. We applied Euclidean distances, which we clustered using Ward's minimum variance method.

**Fig 3:**
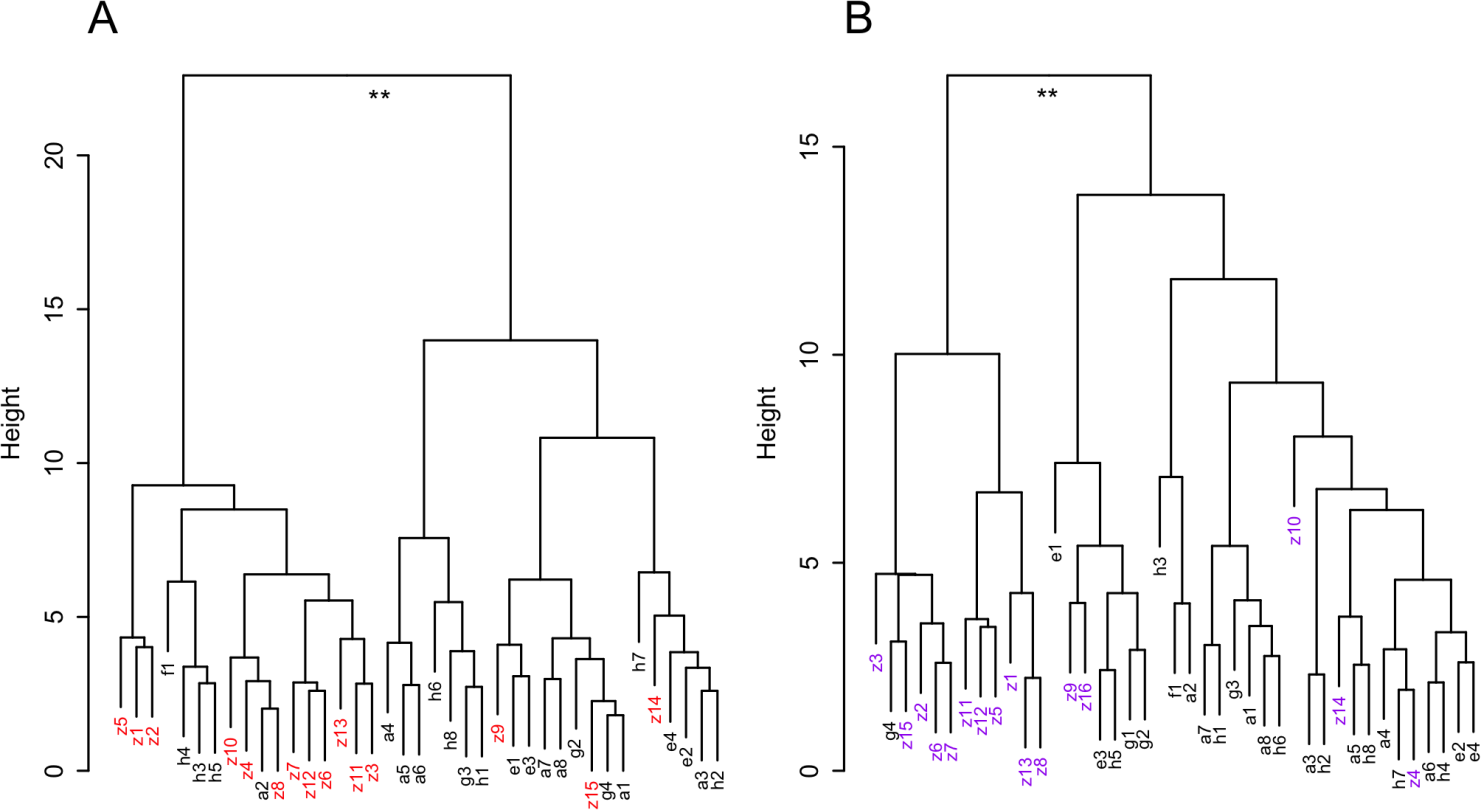
Hierarchical cluster analysis of histone marks. Phenotypes are socialized males (labels a-h, black) and ostracized males (labels z, red or purple) as listed in S1 Table. (A) H3K4me3 marks from 39 mice, (B) H3K27ac marks from 41 mice. Significance of the clusters was tested by a Welch t-test and a permutation test at the deepest split.

The H3K4me3 enrichment data was split into two large clusters (Fig 3A). The mouse identification labels at the leaves of the tree show the phenotype category with letters a to h representing socialized males and z ostracized males (S1 Table). The two clusters formed by the deepest split consisted of 17 and 22 mice, respectively. A permutation test of the phenotype group labels to assess significance of these two clusters resulted in a P-value of 6.2x10^-4^ (mean of three runs) and the Welch test gave a similar result (p = 2.5x10^-4^). In contrast, the two clusters were not separated by body weight (p = 0.86 by permutation and Welch test, S11 Fig). This agrees with our observations from PCA and statistical analyses described above.

The H3K27ac enrichment dataset gave a different tree with one cluster consisting of 12 mice and the second cluster of 29 mice (Fig 3B). At the level of the deepest split, the clustering was significant as tested by permutation (p = 1.47x10^-5^) and the Welch test (p = 2.62x10^-7^). The corresponding tests with mouse body weights did not result in a separation by weight (permutation test p = 0.29 and Welch test p = 0.31, S11 Fig). In summary, the hierarchical cluster analysis agrees with the PCA results: the histone modification enrichment datasets cluster by the two phenotypes but not by body weights.

## Discussion

We show in this study that histone modification patterns at active genes distinguish healthy mice living in nest boxes from badly bitten mice that roam the enclosure. Such socially excluded males are regularly found in enclosure experiments where mice are free to form social structures. The strong social hierarchy observed in mouse colonies forces many males into a subordinate role, and a few males continuously experience social stress. It is interesting to note that we never found any young males outside of a nest box in a similar poor physical state. Because we regularly inspected enclosures and removed all animals, usually males, that showed clear signs of sickness including severe bite marks, we infer that the ostracized males we observed were in this state for no longer than four weeks. We suspect that at least some of these males were former alpha males that had been driven out of the family group by younger, aggressive offspring. Male mice start agonistic behavior when they are around two months old, and often dominant males are superceded by one of their sons that take over the father‘s territory [42]. Since family groups attack strange males, a dominant male that has been driven from its deme will probably not be accepted into another deme [43]. According to the literature on social defeat in male mice, this phenotype developed in the adult animal and is not a condition afflicting these animals from birth or from the prenatal period due to differences in the early environment.

Given this background, we chose ostracized males as a model for studying the effect of social standing on the epigenetic profile of adult tissue. From studies on monozygotic twins it is known that the environment influences epigenetic marks throughout life [44]. Many external stimuli such as lifestyle, seasonal changes, or exposure to toxins have been linked to transcriptional and epigenetic changes, and social interactions are a strong influence on epigenetic settings in specific brain regions [13,15]. Physiological responses to adversity are not restricted to the brain, as is known from the pathophysiological effects of stress on the immune system and its contribution to cardiovascular diseases and metabolic syndrome [45,46]. Moreover, stress responses can be detected as genome-wide changes in DNA methylation in peripheral blood cells [47]. Liver is the largest metabolic organ and given the importance of lipid metabolism, we focused in this study on measuring two histone modifications in liver tissue at genes that are part of the Ppara signaling and lipid metabolism pathways.

Both H3K4me3 and H3K27ac are modifications that are strongly associated with active genes [48–50]. In a three-modification model, the marking of the TSS by H3K27ac was one of the strongest predictors of gene activity in CpG island rich promoters [39]. The TSS marking by H3K4me3 was the best expression predictor in low CpG island promoters [51]. However, our previous genome-wide measurements of H3K4me3 found changes in genes with both promoter types [10]. When we examined the effect of two environments on H3K4me3 marks and gene transcript levels, we found no simple correlation between histone modification and transcription data. Since we concentrated in the present study on the comparison of two histone marks, we restrict the interpretation of our results to epigenetic changes. Our qPCR measurements show that the two markers are correlated but not completely congruent in their response to the social environment of the animals. Recently, H3K27ac has been described as the marker of super-enhancer structures with much wider modification regions than the sharp enhancer peaks [52]. Based on ChIP-Seq data, the wide H3K27ac signal correlated with transcription factor and RNA polymerase II binding patterns. Such super-enhancers might play a critical role in establishing cell type identity by binding master transcription factors [53]. All of our qPCR amplicons were placed well within the peaks of both H3K4me3 and H3K27ac as we show by way of example for three loci in the UCSC genome browser tracks of S2 Fig. It is therefore unlikely that the measured differences between H3K4me3 and H3K27ac plasticity are the result of sub-optimal amplicon position for one of the markers. On the contrary, the differences in H3K4me3 and H3K27ac responses, such as the reverse marking at *Slc27a5* and *Pparg*, are possibly due to functional differences between the two modifications. In embryonic stem cells H3K27ac super-enhancer sites regulate genes that define the cell type [52]. In a tissue containing almost only differentiated cells, the H3K4me3 modification may be more sensitive to hormonal or intracellular signals than H3K27ac. Since most of the research linking histone modifications to transcription levels is performed on cell cultures and measures changes in the course of differentiation rather than subtle metabolic shifts of an intact tissue in response to varying demands on homeostatic control mechanisms, the present study on adult mouse liver is one of the few examples for quantifying epigenetic plasticity under physiological conditions. Expanding of our study by measurements of other epigenetic marks and of transcription levels would allow us to investigate the relationship between epigenetic changes and transcription levels in a real tissue.

We applied three types of analyses to our enrichment data: tests of equality of means and variances, PCA, and hierarchical clustering. The Manova analysis divided both datasets into socialized and ostracized mice, but not into light and heavy mice. Using a per locus analysis, we extracted the individual genes where enrichment patterns changed significantly. The glucocorticoid receptor *Nr3c1* is a particularly relevant locus in our study because changes in its DNA methylation and gene activity have previously been observed under social stress in brain tissue. For example, expression levels were upregulated in hippocampus tissue from mice that had experienced early life stress [54]. In liver, *Nr3c1* expression was increased and DNA methylation was decreased in newborn rats when the mother had been fed a protein restricted diet [34]. In that study, *Ppara* was also upregulated, which is similar to the upmarking of both loci in the ostracized males. However, our experimental setup is quite different from such dietary studies. As a result, we measured a decrease of activating histone marks at the genes for the enzymes fatty acid synthase (*Fasn*) and phosphoenolpyruvate carboxykinase (*Pck1*), which contrasts with the diet studies. Nevertheless, our data suggests that the transcript and hypomethylation changes that are the focus of many epigenetic studies in mice could be complemented by the quantification of histone modifications as these also respond to environmental changes in a similar way.

At two loci, *Fasn* and *Cyp4a14*, histone enrichment between socialized and ostracized males was statistically significant for one marker but not for the other one. Since the mean values at both loci were smaller in the ostracized males, larger samples could help to resolve such differences between the two histone markers. Another way to corroborate the effects would be to use a series of PCR products within one histone modification peak. The generalization of this approach would be a genome-wide search for histone modification differences by ChiP-Seq, as was carried out in our previous study on quantitative differences in H3K4me3 modification [10]. Alternatively, a genome-wide analysis of differentially methylated sites could be used to confirm histone modification results. However, studying DNA methylation might limit the findings to differences in genes with CpG-rich promoters, which characterize house-keeping genes [55]. For example, a recent genome-wide survey of differentially methylated regions in obese versus lean humans highlighted primarily developmental genes and those connected to transcription and cell differentiation [56]. Only when the authors used a list of candidate obesity genes from genome-wide association studies, did they find a significant enrichment of genes linked to obesity and related diseases among differentially methylated loci.

After calculating P-values, we visualized the separation of the datasets and controlled for other possible groupings. PCA and hierarchical clustering use different aspects of a dataset for classification. PCA transforms a set of variables that may be correlated into linearly uncorrelated components. A complex dataset is thus reduced to synthetic variables that sequentially account for variance-components. For example, PCA on CpG island methylation data from mouse liver partially separated an obese phenotype from controls [57]. In this study, the first PC accounted for 22% of data variance and the first four PCs for over half of the variability of the dataset. The DNA methylation data of treatment versus control substantially overlapped in the PC1—PC2 plot, but a visual separation was present at PC3 and PC4. In our histone modification analysis, the variance explained by PC1 was slightly greater (24 to 25%), as was the sum of PC1 to PC4 with 67% (H3K4me3) and 68% (H3K27ac). Moreover, ostracized mice were separated from socialized mice along PC1 in the H3K4me3 dataset and along PC2 in the H3K27ac dataset. Mice sampled from different nest boxes, however, did not separate by PCA, suggesting that genetic diversity, which is usually lower within than between nests [2,58], did not play a role in the histone modification variation we measured. These results are preliminary, as our data is restricted to a small number of genes, but genome-wide comparisons of epigenetic patterns in outcrossing mice would allow us to study whether histone modification differences between individuals are mostly due to the environment or are also determined by genetic factors.

PCA was also a suitable method to test whether factors other than social status separate the mice. Body weight and age might by themselves trigger changes in epigenetic settings. Since our mice stem from a free-living population rather than having been reared in a cage, where social interactions are restricted, we were not able to disentangle social effects from age. Our wild mice reach sexual maturity at around 12–13 g of body weight (Table 1); thus by choosing males over 20 g, we selected mice that had developed well into adulthood. However, we used body weight as a second factor in the analysis. For H3K4me3, we detected no significant correlation of the PC separating social status with body weight, and the PC plots where mice were color-grouped by body weight did not result in separation either (S5 Fig). In contrast, the H3K27ac modification was not independent of body weight, because the PC plots partially grouped mice weighing either under or over 25 g (S6 Fig). The strong difference at *Plin5* was present in both phenotype and weight comparisons (Table 3). In the H3K4me3 dataset we did not find any locus where these two comparisons were both highly significant, and this might be the reason for the difference in weight dependence between the two markers. However, since the liver plays a central role in lipid transport and metabolism, liver gene activity may be influenced by body weight and vice versa. The liver produces bile salts that are necessary for the uptake of dietary fat in the gut, and secretes lipoproteins to transport triglycerides and cholesterol in the blood stream to adipose tissue and muscle. Liver cells break down dietary fatty acids into acetyl-CoA, but also synthesize endogenous fatty acids and triacylglycerols that can be exported to adipose tissue. Liver lipid metabolism is regulated by central signals through innervation and hypothalamic neuropeptides [59]. For example, neuropeptide Y activates triglyceride secretion [60]. The liver may also respond directly to the satiety hormone leptin via its own leptin receptor, which regulates lipoprotein remodeling and increases lipoprotein lipase activity in a mouse model [61]. A direct link between liver gluconeogenesis and body weight has recently been proposed through the regulatory enzyme fructose-1,6-bisphosphatase, which, when overexpressed, decreased body weight by the downregulation of appetite stimulation [62,63]. Because of the complex interactions between central and peripheral signals that regulate energy homeostasis, an influence of body weight on histone modification differences as observed by us in S6 Fig is not surprising. We have obtained similar results for H3K4me3 in a pilot study where we compare young females (16–20 g body weight) with older females (21–32 g body weight). These two groups were not separated by PCA (S12 Fig), but the same sample was split by the two environmental conditions imposed on the mice [10]. This suggests that, in adult mice, a strong difference in the environment can have a larger effect on liver histone marks than body weight.

The pairwise Euclidean distances that we used in the hierarchical clustering analyses also resulted in a clear and significant separation of ostracized from socialized mice. However, the two histone modifications H3K4me3 and H3K27ac show subtle differences in clustering. For example, the deepest split of the H3K4me3 tree divides the sample into two groups of equal size whereas the deepest split of the H3K27ac tree forms two unequal sets that are very similar to the true sample composition containing fewer ostracized mice. Taken together, these three analysis methods agree in splitting the mouse sample along the social experience of the animals. We conclude that the social position of male mice can lead to quantitative alterations in liver histone modifications. The data presented in this study come from a single experiment which involved a population of approximately 400 mice. To enhance reproducibility, we extended a pilot sample of 24 mice, where we already found the described effects [64], by another 17 mice. Ideally, one would like to sample mice from a second, independent population to test whether the effect of social standing on histone marks can be repeated in another cohort of animals. In addition, it would be interesting to conduct a genome-wide search for differences in histone modifications to see which other genes or functional pathways are involved in upholding metabolic homeostasis after the experience of social stress.

## Methods

### Ethics statement

The study was carried out in accordance with German animal welfare legislation. Animal work was registered under V312-72241.123-34(97-8/07) and approved by the ethics committee of the Schleswig-Holstein State Ministry for Agriculture, Environment and Rural Areas. House mice are progenies of individuals captured in Germany in 2007. Permissions and procedures have been described previously in detail [10,65].

### Enclosure population of outcrossing house mice

Our wild house mouse population (*Mus musculus domesticus*) descends from mice originally caught in stables and barns around Cologne and Bonn, Germany, and kept in the laboratory under an outbreeding scheme [66]. As reported recently [10], we started a mouse population in an enclosure of 18 m^2^ with 10 mature females and males each. Mice were provided with an enriched environment similar to classical experiments [2,6]. Each of the two enclosures contained ten nest boxes, spacial structures (wooden boards and plastic tubes), nesting material, and food (Altromin 1420) and water *ad libitum*. Founder animals were removed once the first generation born in the enclosure reached sexual maturity. Mice were inspected daily. Every 4–6 weeks, all mice were trapped by either closing the nest boxes or in live-traps to monitor the population. Mice were counted, weighed, and their health status was assessed by inspecting their fur and searching for bite wounds and ear mites.

The population of enclosure B [10] grew rapidly due to an energy-enriched diet and longer day hours that mimicked summer conditions. The mice of the present study were from this crowded population and were sampled when the population was 8 months old. A total of 41 males were selected for the epigenetic analysis of liver tissue. They weighed more than 20 g and were hence judged to be at least 6 weeks old. Social status was assessed by location, i.e. in a nest box or freely roaming, and by the condition of the fur, i.e. healthy or wounded (S1 Table). Mice were dissected and liver samples were snap frozen in liquid nitrogen, and stored at -80°C.

### Chromatin preparation and immunoprecipitation

Soluble chromatin was prepared from liver tissue stored at—80°. Tissue pieces of 100 to 180 mg were thawed on ice and, after addition of 1 ml of phosphate buffered saline (PBS) containing 1% of the cross-linking agent formaldehyde, cut into pieces of 1 mm^3^ with a pair of fine scissors. After incubation for 10 min, samples were briefly centrifuged, the supernatant was discarded and 1ml of 125 mM glycine in PBS was added to the tissue pellet to stop the cross-linking reaction. After incubation for 10 min on a rotating wheel, samples were centrifuged and the tissue pieces were washed twice in cold PBS containing 1mM PMSF. Pellets were resuspended in lysis buffer (SimpleChip Enzymatic Chromatin IP Kit, Cell Signaling) and were homogenized on ice in a 5 ml Dounce homogenizer with 10 strokes using pestle A and 10 strokes using pestle B. Samples were left on ice for at least another 10 min. To cut chromatin into nucleosome fragments, lysates were incubated with micrococcal nuclease for 20 min at 37°C and sonicated 3 to 4 times for 30 sec on ice with 1 min resting intervals at an amplitude of 70–80%. Insoluble material was removed by centrifugation at 10,000 rpm for 10 min at 4°C, the soluble chromatin was shock frozen on dry ice and kept at -80°C. DNA concentration in the chromatin preparation was measured from aliquots using the Quant-iT dsDNA Broad-Range Assay Kit (Life Technologies) and a NanoDrop 3300 Fluorospectrometer.

Input control samples corresponded to 2% of the lysates that were subjected to immunoprecipitation. They were taken from pre-cleared chromatin preparations and kept at -20°C. Immunoprecipitations were performed on 1 μg of chromatin per sample in a volume of 300 μl and 2 μl of Tri-Methyl-Histone H3 (Lys4) (C42D8) rabbit monoclonal antibody or Acetyl-Histone H3 (Lys27) Antibody (Cell Signaling, #9751 and #4353, respectively). Negative controls were prepared with 0.5 μl of rabbit IgG (Cell Signaling, #2729). Samples were incubated at 4° C over night on a rotating wheel and ChIP-Grade Protein G Agarose beads (Cell Signaling, #9007S) were added for at least another 2 h to capture the beads. Immunoprecipitates were collected by brief centrifugation at 10,000 rpm and resuspended in 1 ml of ice-cold low salt ChIP-wash buffer. This was repeated another two times followed by a final wash in high salt ChIP wash buffer. The wash solution was carefully removed, beads were resuspended in 150 μl of elution buffer, and incubated at 65°C for 30 min on a rotating platform (1,200 rpm). To digest histone and antibody protein, supernatants were treated with 2 μl of proteinase K (20 mg/ml) at 65°C for 2 h, and DNA was purified on spin columns (MinElute PCR Purification Kit, Quiagen) by two consecutive elutions with 35 μl of TElow buffer (10 mM Tris, pH 8, 0.1 mM EDTA). DNA was kept at -20°C before further processing.

### qPCR

Samples of ChIP-DNA, input and negative controls (70 μl) were mixed with 250 μl Fast Sybr Green Master Mix (Applied Biosystems), and 130 μl pure water (HPLC standard). qPCR measurements were performed in triplicates. Primers (1 μl) were distributed on 96 well plates, 9 μl of the DNA-Sybr-Green mixture was added to each well, and the plate was sealed with a thermal foil. DNA was quantified on a 7900HT Fast Real-Time PCR System (Applied Biosystems) and the PCR reaction was run with temperature cycles of 20 sec at 95°C for initial denaturation, and 40 cycle repeats of 1 sec at 95°C for denaturation and 20 sec at 60°C for annealing and extension. The fluorescence signal was turned into cycle at threshold (Ct) values using the Applied Biosystems SDS 2.3 software and the automatic threshold setting.

Primer sequences, melting temperatures, and expected amplicon sizes are given in S5 Table. Primers were tested on genomic DNA (chromatin before immunoprecipitation) from two C57Bl/6 and two wild mice. PCR reactions resulted in one primer product of the expected sizes as seen on 2% agarose gels (S13 Fig). In some preparations, there was a second, smaller band visible at loci Slc27a5 and Acox2; this was observed from both inbred and wild mouse preparations in end product solutions after 40 cycles of qPCR. To calculate primer efficiencies, qPCR measurements were performed from a 1:10 dilution series that corresponded to 20%, 2%, and 0.2% of the input. Efficiencies were calculated from the slope of a regression line with Ct values on the y-axis and DNA dilutions on the log10 x-axis. With the exception of three primer pairs, primer efficiencies were within the efficiency range of 1.9 to 2.1 (S5 Table). When efficiencies for *Cd36*, *Slc27a5*, and *Acox2* were calculated from the 2% and 20% input Ct values, which also corresponded best with the measurement values, primer efficiencies decreased from 2.2–2.4 to 2.1–2.2. We therefore used an efficiency of 2.0 for the data calculation throughout. Fold enrichment over rabbit IgG varied between 1.6 and 97 in H3K4me3 immunoprecipitates and between 1.3 and 7.2 in H3K27ac immunoprecipitates (S14 Fig). We omitted *Cd36* in the data discussion because the fold enrichment over negative control was less than two.

Enrichment over input was calculated by the delta Ct method (input—immunoprecipitate). We normalized the datasets by two methods, first against *Gapdh* (enrichment = 1), and second against the mean of all loci (mean enrichment over 15 loci = 1). Normalization by just one locus may lead to a distortion of the results in case the control locus behaves atypically. Vandesompele [30] suggests normalization with respect to nine housekeeping genes in expression data analyses. Because our chromatin DNA yield from tissue samples was not high enough to allow several house-keeping gene controls, we used the arithmetic mean of all 15 loci. This normalization method has the advantage that it centers the datasets at enrichment value 1 and allows an easier numeric comparison between the groups or between the two epigenetic markers.

Of the 41 mice selected for analysis, two samples had large standard deviations in the mean-normalized H3K4me3 qPCR dataset. They are marked in S1 Table and were omitted from the H3K4me3 data analysis unless stated otherwise. Preparation 1 of ChIP and qPCR (S1 Table) was originally a project using 24 mice [64]. To increase the number of samples and to repeat the outcome of a complex laboratory protocol, another member of the lab independently prepared 17 more samples (preparation 2).

### Statistical analysis

To describe variation within a dataset, we calculated the coefficient of variation (standard deviation divided by mean). Since our data may not be normally distributed, we assessed the significance of differences in means or variances by a permutation test that shuffled group labels with 10,000,000 iterations. Given is the mean P-value from three runs. To correct for testing at multiple loci, we used the Benjamini-Hochberg method and adjusted raw P-values by the "p.adjust" function in R. F-tests and t-tests to assess significance of enrichment data at 15 individual loci were performed on Excel Spreadsheets. We used a two-sided Student's t-test for equal variances and the Welch two sample t-test for unequal variances. Anova, Manova, PCA and hierarchical clustering analyses were performed in R. Manova was performed on 14 loci to avoid dependencies due to the normalization by using the average over all 15 loci. In the H3K4me3 dataset, *Cd36* was omitted because the mark was near zero. In the H3K27ac dataset, *Gapdh* was omitted as the weakest enrichment data point (Fig 1). To be consistent, PCA and cluster analyses were performed on data from the same 14 loci. The hierarchical cluster analysis used as distance function the square distance between the two vectors (argument method = "euclidean") and as agglomeration method Ward's minimum variance method (argument method = "ward"). Input data were centered and scaled data matrices were obtained from the R function "scale(data)" with default settings.

The present study focuses on the comparison between the two phenotypes "socialized" and "ostracized" males. Since body weight might contribute to quantitative changes in histone modifications, we contrasted the phenotype comparisons with those of comparing light with heavy mice. For this test, we selected a) two equally sized groups of light and heavy mice, and b) two smaller groups with the lightest and heaviest mice. An important control was also the comparison between two nests (socialized males only). In addition, we checked whether sample preparation influenced histone mark changes and used a) only socialized mice, b) only ostracized mice, or c) an even mixture of both.

## Supporting Information

S1 Fig H3K4me3 and H3K27ac enrichment over input. (A,B) Data were normalized against the mean of all loci and correspond to Fig 1. (C,D) Data were normalized against *Gapdh*. H3K4me3 data are shown in (A,C), H3K27ac data in (B,D). Labels refer to mouse IDs shown in S1 Table.
(TIF)

S2 Fig Position of qPCR amplicons within H3K4me3 and H3K27ac ChIP-Seq peaks. The positions of qPCR amplicons used in this study were determined by in-silico PCR on the UCSC mouse genome browser platform (July 2007, NCBI37/mm9 assembly). Traces in black are a custom browser track from our mouse population [10]. Yellow traces are from ENCODE/LICR for H3K4me3 (DCC accession wgEncodeEM001444) and H3K27ac (DCC accession wgEncodeEM002500) obtained from adult mouse liver. (A) TSS of gene *Gapdh*, (B) TSS of gene *Acox2*, (C) TSS of gene *Cyp4a14*.
(TIF)

S3 Fig Principal components analysis of H3K4me3 enrichment testing phenotype. Enrichment data from 39 mice and 14 loci are used for PCA. Plots show the contributions of PC1 to PC5 in the PCA. Colors highlight the two phenotypes: black are socialized males (n = 24), and red are ostracized males (n = 15).
(PDF)

S4 Fig Principal components analysis of H3K27ac enrichment testing phenotype. Enrichment data from 41 mice and 14 loci are used for PCA. Plots show the contributions of PC1 to PC5 in the PCA. The two phenotypes are highlighted by color: black are socialized males (n = 25), and purple are ostracized males (n = 16).
(PDF)

S5 Fig Principal components analysis of H3K4me3 enrichment testing the effect of body weight. Enrichment data from 39 mice and 14 loci were used for PCA. Mice were assigned by body weight into three groups: black 20.0–22.4 g (n = 13), grey 22.5–24.9 g (n = 14), and green 25.0–30.0 g (n = 12). Plots show the contributions of PC1 to PC5.
(PDF)

S6 Fig Principal components analysis of H3K27ac enrichment testing the effect of body weight. Enrichment data from 41 mice and 14 loci were used for PCA. Mice were assigned by body weight into three groups: black 20.0–22.4 g (n = 14), grey 22.5–24.9 g (n = 14), green 25.0–30.0 g (n = 13).
(PDF)

S7 Fig Principal components analysis of H3K4me3 enrichment after *Gapdh* normalization. The dashed line is drawn through the origin of the plot PC1 vs. PC2 with a slope of -1 and shows the group separation that has been used for the permutation test. Three permutation runs of this separation by PC1 and PC2 gave a mean P-value of 3.1x10^-3^. When the dataset was divided into two groups at the level of PC1 = 0, this separation was marginally significant (p = 4.8x10^-2^), but grouping at the level of PC2 = 0 did not give a significant result (p = 9.8x10^-2^).
(PDF)

S8 Fig Principal components analysis of H3K27ac enrichment after *Gapdh* normalization. The dashed line is drawn through the origin of the plot PC2 vs. PC3 with a slope of +1 and shows the group separation that has been used for the permutation test. Three permutation runs of this separation by PC2 and PC3 gave a mean P-value of 1.1x10^-5^. When the dataset was divided into two groups at the level of PC2 = 0, the mean P-value was 2.2x10^-2^; when it was divided at the level of PC3 = 0, the mean P-value was 1.4x10^-3^.
(PDF)

S9 Fig PCA of enrichment data comparing 16 mice from two nest boxes. The percentage is the fraction of variance that is explained by the respective PC. Blue are mice from nest A, orange are mice from nest H. (A, B) H3K4me3 enrichment data, (C, D) H3K27ac enrichment data. (B) The dashed line shows a visual separation that splits the sample into two groups of 8 individuals each. This separation was tested by permutation and was found not to be significant (mean P-value of three runs: 0.13).
(PDF)

S10 Fig PCA of enrichment data comparing two chromatin-immunoprecipitation preparations. (A, B) H3K4me3 data of 39 mice were analyzed. (C, D) H3K27ac data of 41 mice were analyzed. Samples from preparation 1 are pink, samples from preparation 2 are cyan. (A) The significance of a possible separation of H3K4me3 data along PC2 = 0 was tested by permutation. The mean P-value of three runs was 5.5x10^-2^.
(PDF)

S11 Fig Hierarchical cluster analysis of H3K4me3 and H3K27ac enrichment data at 15 loci. The analysis was performed in (A) from the H3K4me3 dataset with 39 mice (24 mice socialized, 15 mice ostracized) and in (B) from the H3K27ac dataset with 41 mice (25 mice socialized, 16 mice ostracized). Leaf labels are body weight in g. The mean body weights of the two largest clusters were 23.9 ± 2.3 and 23.7 ± 2.5 (mean ± sd in A) and 24.4 ± 2.5 and 23.5 ± 2.3 (in B). Means were not different as determined by a Welch two sample t-test and a permutation test.
(TIF)

S12 Fig Comparison of H3K4me3 marks from younger and older females. H3K4me3 qPCR enrichment data from 24 healthy females that lived in two different environments [10] were analyzed by PCA. Grey are young females living under standard conditions (body weight 17–20 g) and black are older females from the same environment (22–32 g). Orange are younger females living in a summer environment with an energy-enriched diet (body weight 16–18 g) and brown are older females from the same environment (21–30 g). The 15 gene loci of this dataset were *Gapdh*, *Cdkn1a*, *Cyp2j5*, *Igf1*, *Igfbp2*, *Insig2*, *Plin5*, *Pop4*, *Ppara*, *Rpp21*, *Rsl1d1*, *Serpina6*, *Slc38a3*, *Smtn*, and *Tgfbr2*. The two different environments had a stronger effect on group separation than the age of the animals.
(PDF)

S13 Fig Control gel of PCR products. PCR products from one of the wild mouse chromatin preparations were separated on a 2% agarose gel and visualized using Sybr Green under UV. All PCR products were at the correct position according to the calculated amplicon lengths.
(TIF)

S14 Fig Fold enrichment of immunoprecipitates over negative control. Boxplots show mean fold enrichment values of H3K4me3 and H3K27ac immunoprecipitates over rabbit IgG immunoprecipitates obtained from 41 samples. The ranking within the set of 15 loci is different between H3K4me3 and H3K27ac immunoprecipitates.
(TIF)

S1 Table Body weights and fur condition of mice analyzed in this study. (DOCX)

S2 Table qPCR data analysis: means and standard deviations of two normalization methods. (DOCX)

S3 Table Comparison of P-values from permutation and conventional statistical tests. (DOCX)

S4 Table Manova analyses of H3K4me3 and H3K27ac enrichment data from socialized and ostracized mice. (DOCX)

S5 Table Primer sequences for the qPCR analysis. (DOCX)
