# Supplementary figures and images for: Social Exclusion Changes Histone Modifications H3K4me3 and H3K27ac in Liver Tissue of Wild House Mice
Source: PLoS One. 2015 Aug 12;10(8):e0133988. doi: 10.1371/journal.pone.0133988 (PMC4534140; doi:10.1371/journal.pone.0133988)

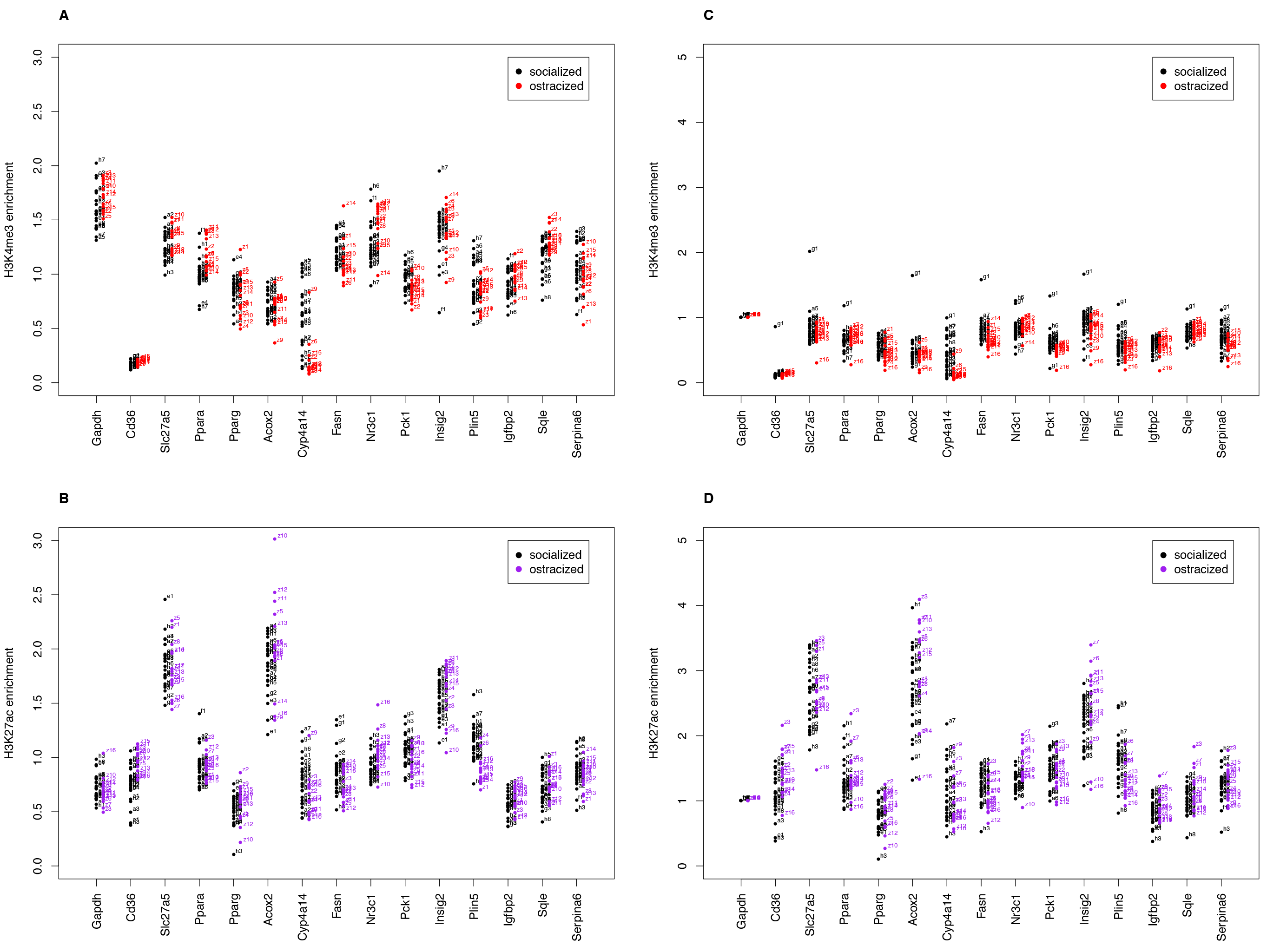

Supplement: S1 Fig — (A,B) Data were normalized against the mean of all loci and correspond to Fig 1. (C,D) Data were normalized against Gapdh. H3K4me3 data are shown in (A,C), H3K27ac data in (B,D). Labels refer to mouse IDs shown in S1 Table. (TIF) [file pone.0133988.s001.tif]

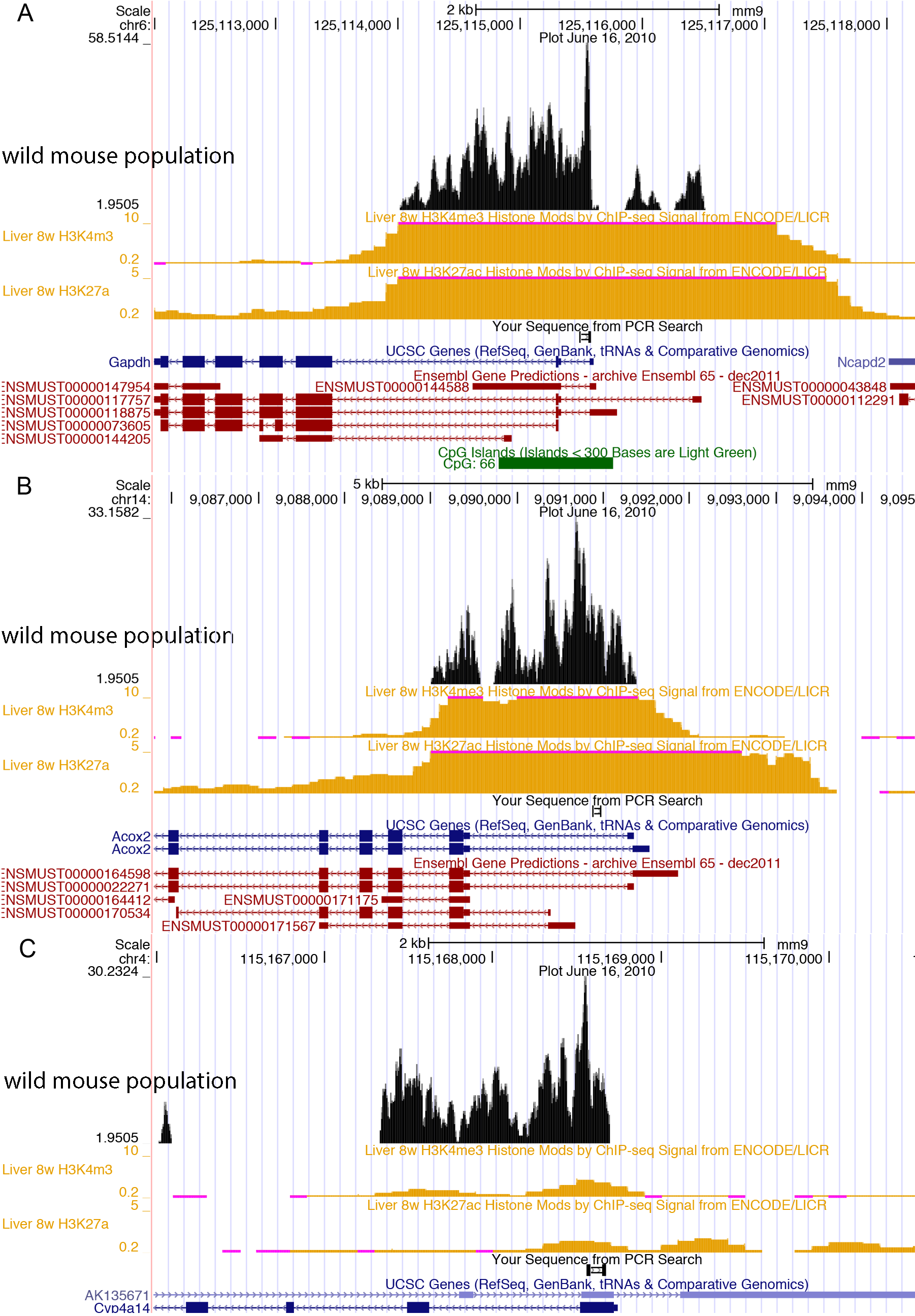

Supplement: S2 Fig — The positions of qPCR amplicons used in this study were determined by in-silico PCR on the UCSC mouse genome browser platform (July 2007, NCBI37/mm9 assembly). Traces in black are a custom browser track from our mouse population [10]. Yellow traces are from ENCODE/LICR for H3K4me3 (DCC accession wgEncodeEM001444) and H3K27ac (DCC accession wgEncodeEM002500) obtained from adult mouse liver. (A) TSS of gene Gapdh, (B) TSS of gene Acox2, (C) TSS of gene Cyp4a14. (TIF) [file pone.0133988.s002.tif]

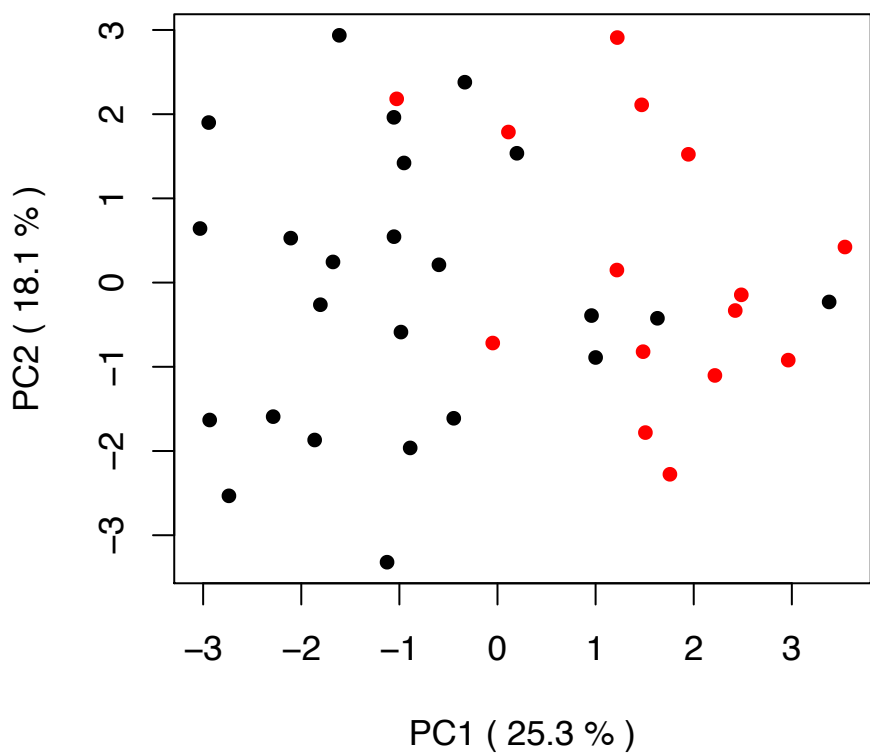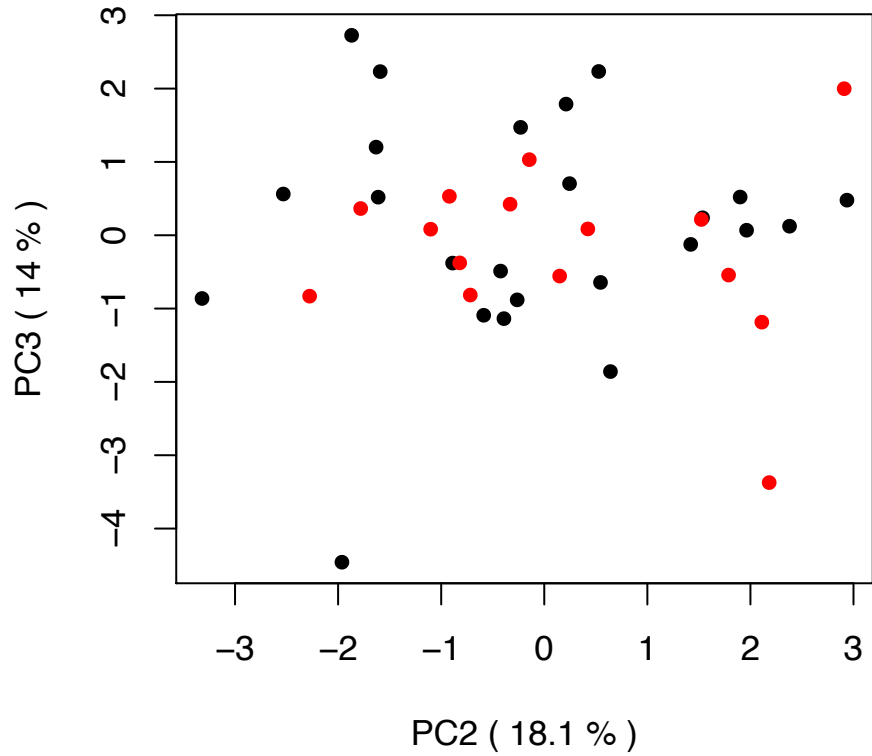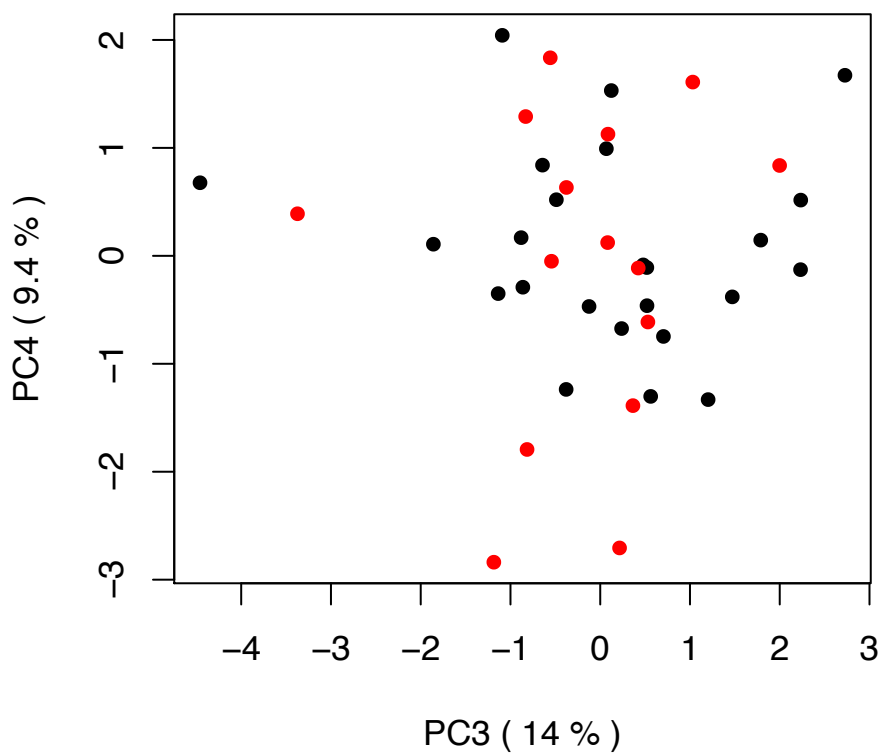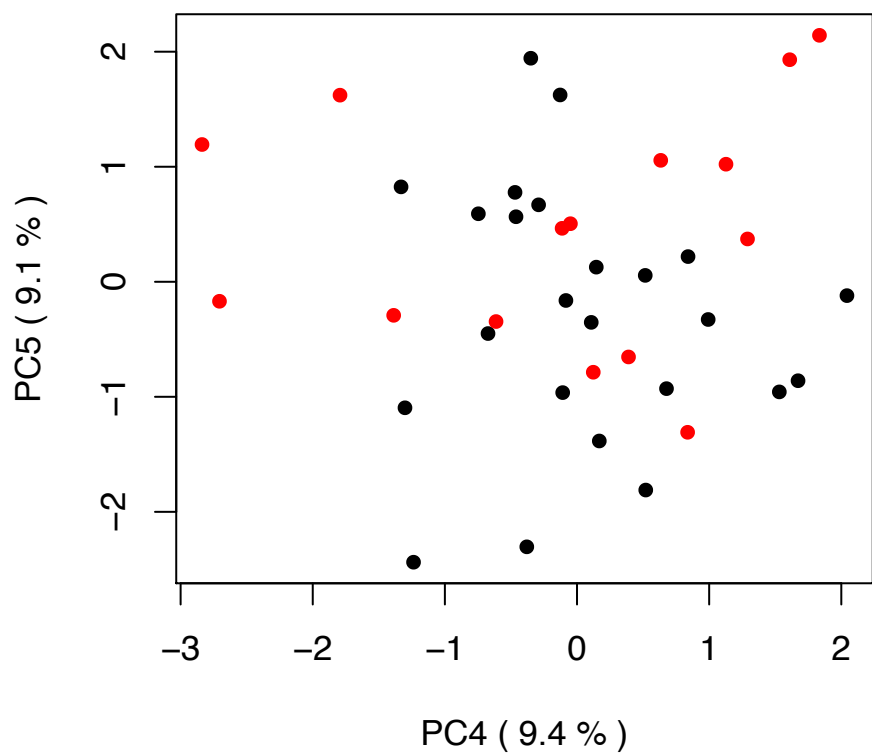

Supplement: S3 Fig — Enrichment data from 39 mice and 14 loci are used for PCA. Plots show the contributions of PC1 to PC5 in the PCA. Colors highlight the two phenotypes: black are socialized males (n = 24), and red are ostracized males (n = 15). (PDF) [file pone.0133988.s003.pdf]

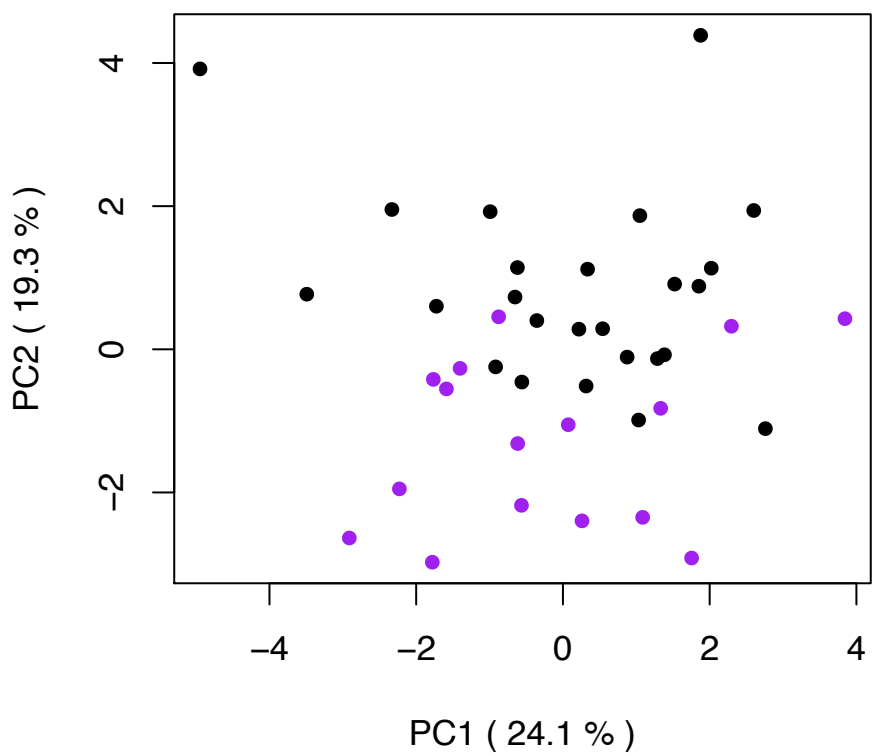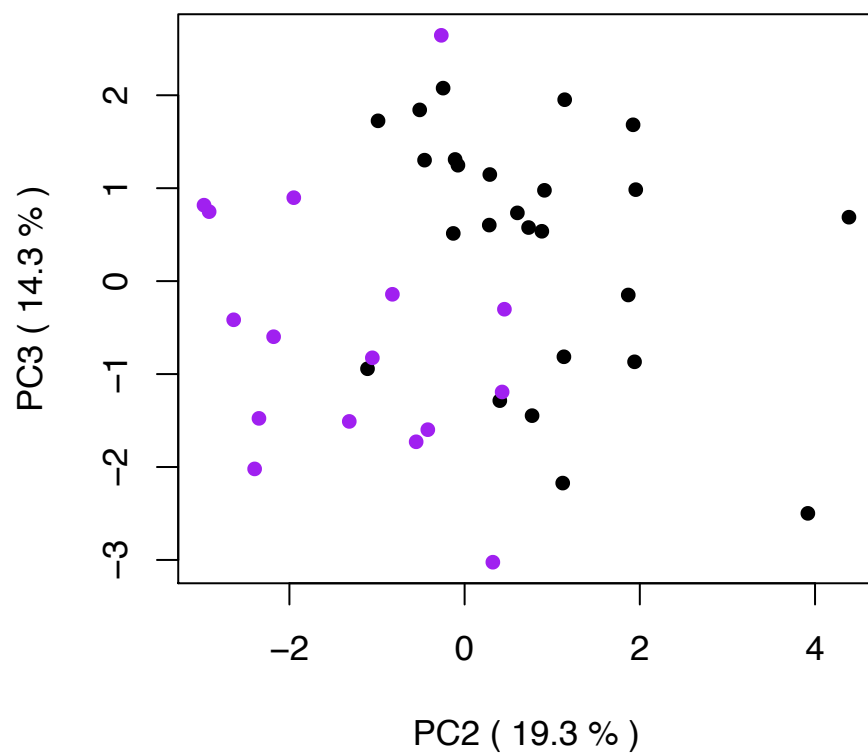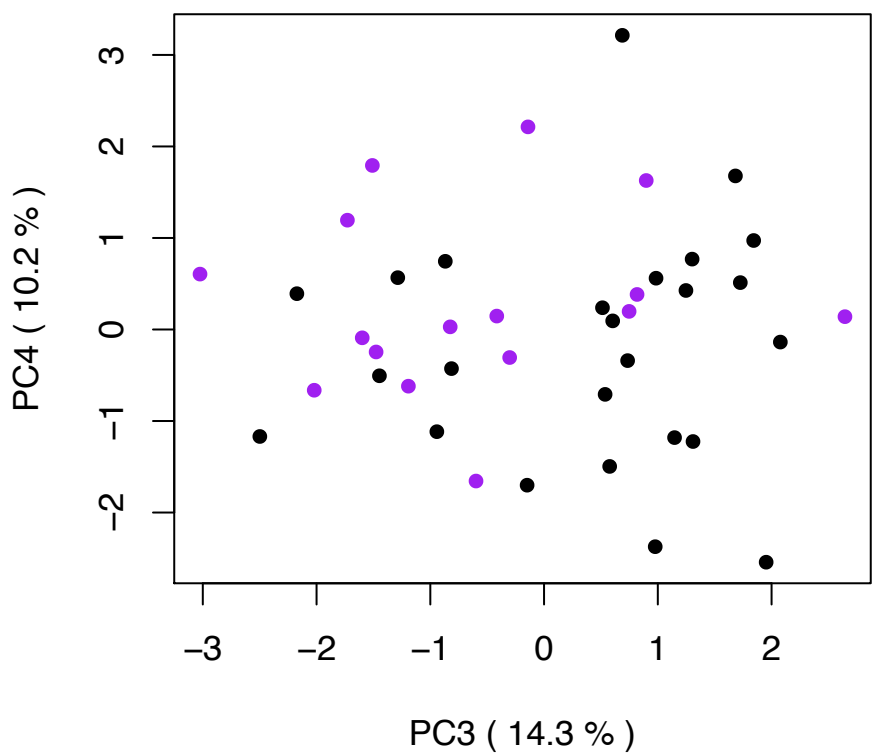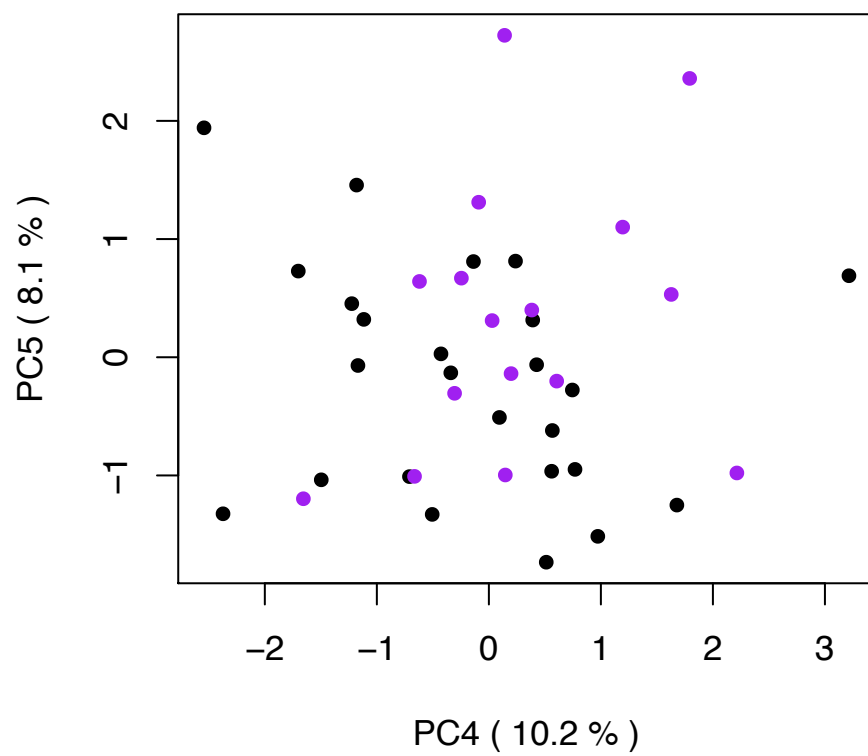

Supplement: S4 Fig — Enrichment data from 41 mice and 14 loci are used for PCA. Plots show the contributions of PC1 to PC5 in the PCA. The two phenotypes are highlighted by color: black are socialized males (n = 25), and purple are ostracized males (n = 16). (PDF) [file pone.0133988.s004.pdf]

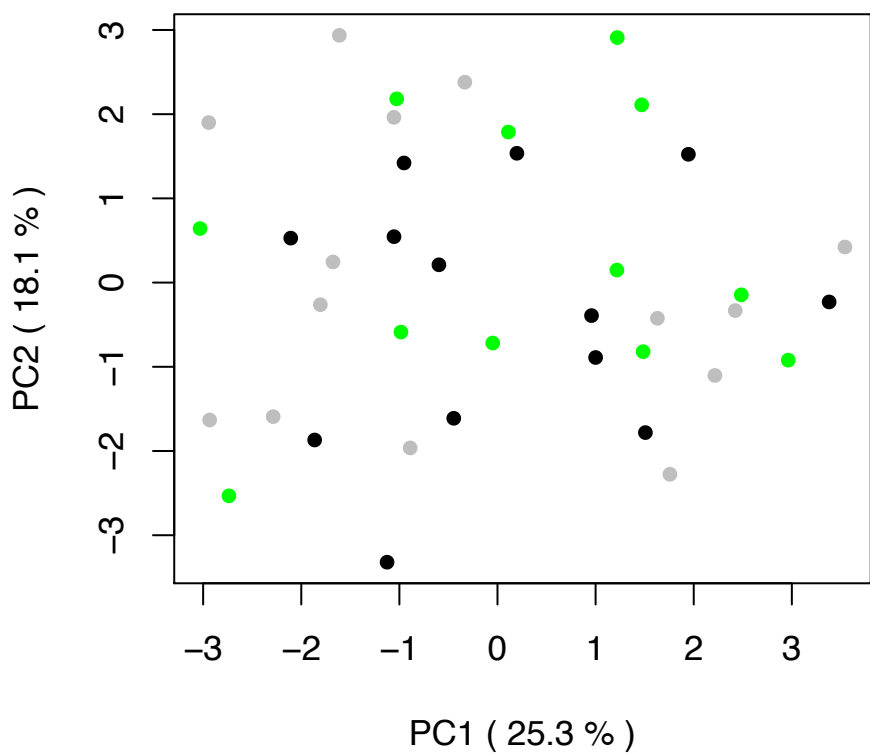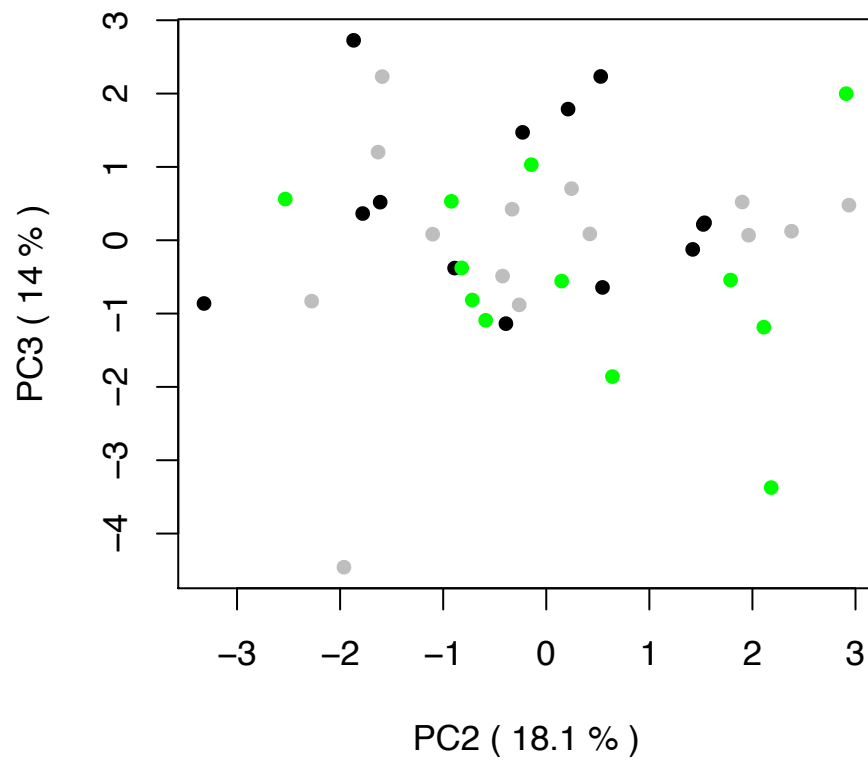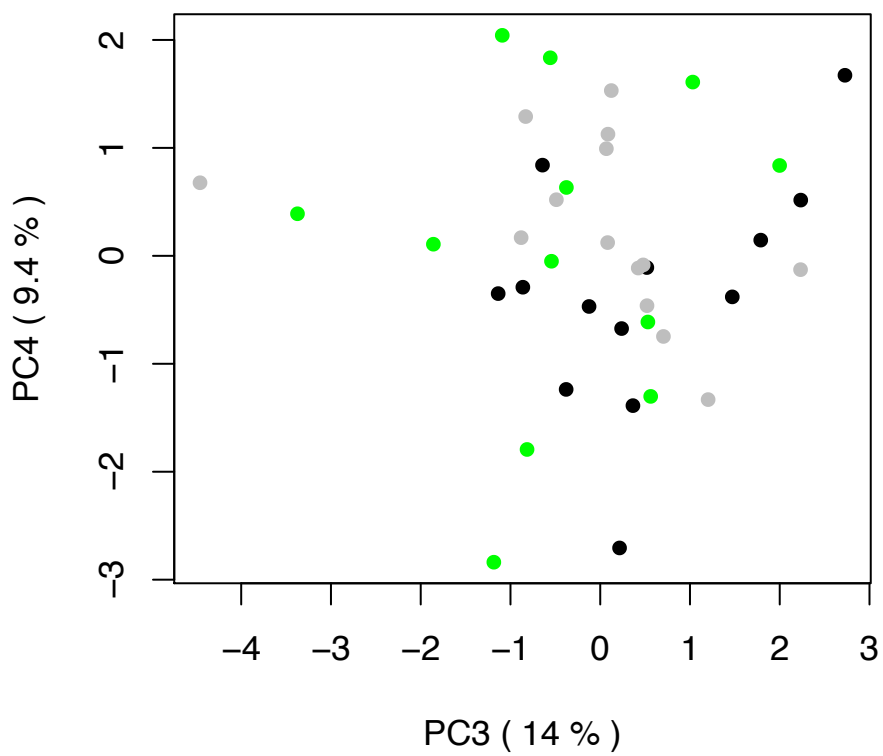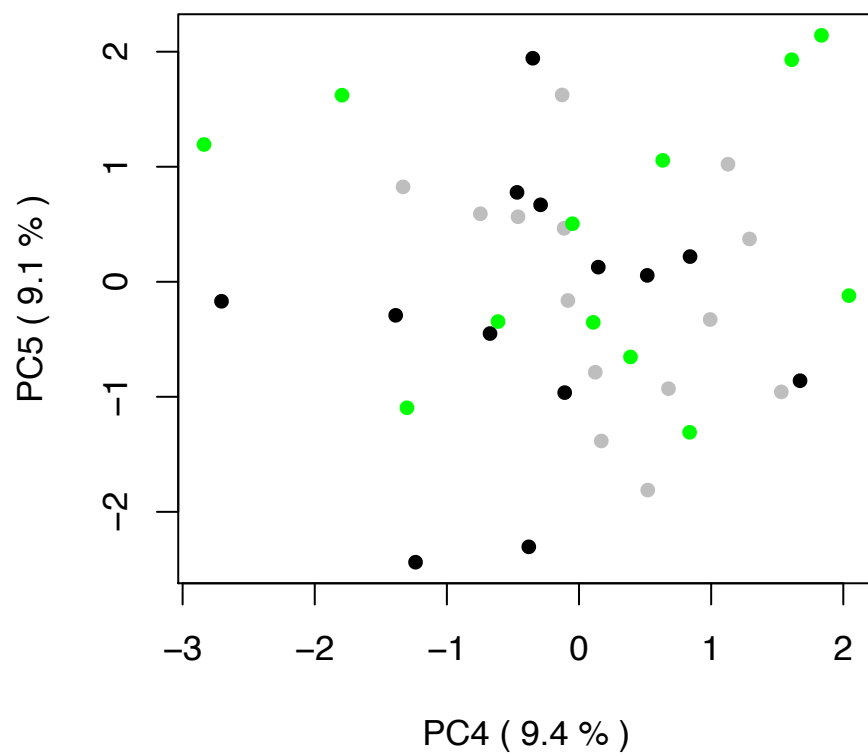

Supplement: S5 Fig — Enrichment data from 39 mice and 14 loci were used for PCA. Mice were assigned by body weight into three groups: black 20.0–22.4 g (n = 13), grey 22.5–24.9 g (n = 14), and green 25.0–30.0 g (n = 12). Plots show the contributions of PC1 to PC5. (PDF) [file pone.0133988.s005.pdf]

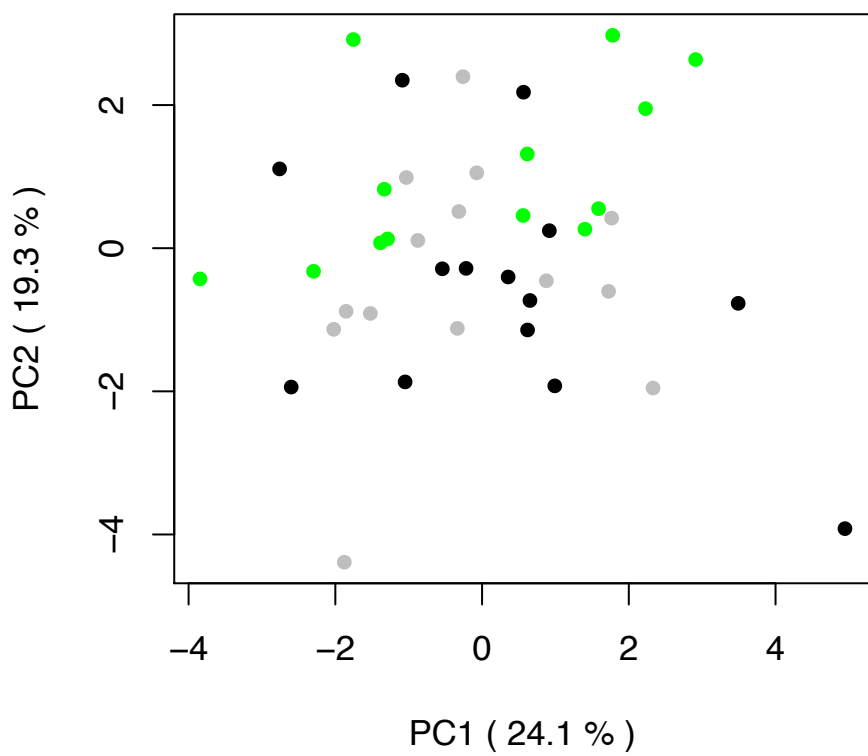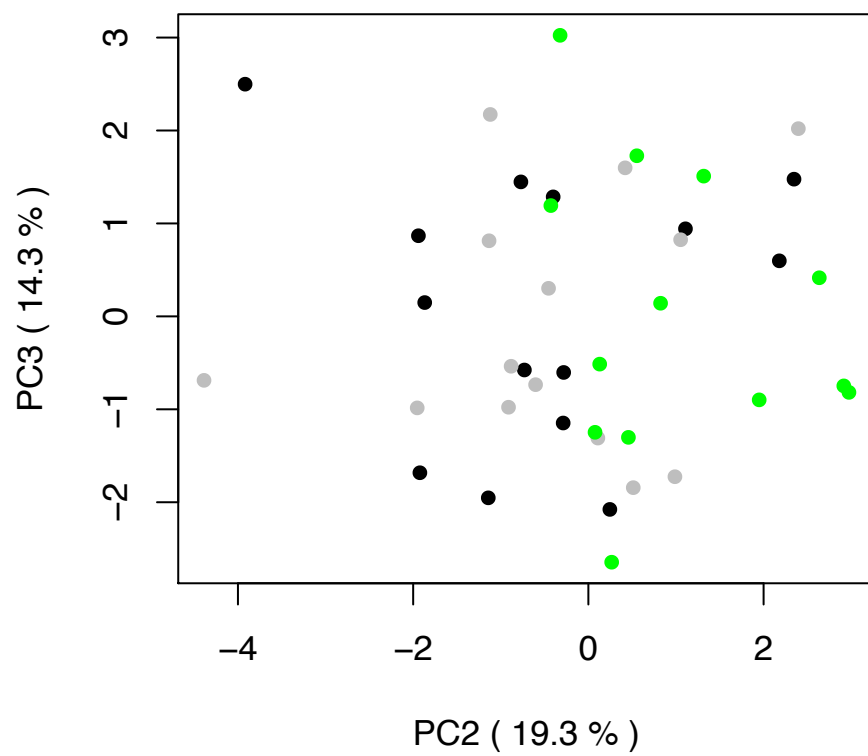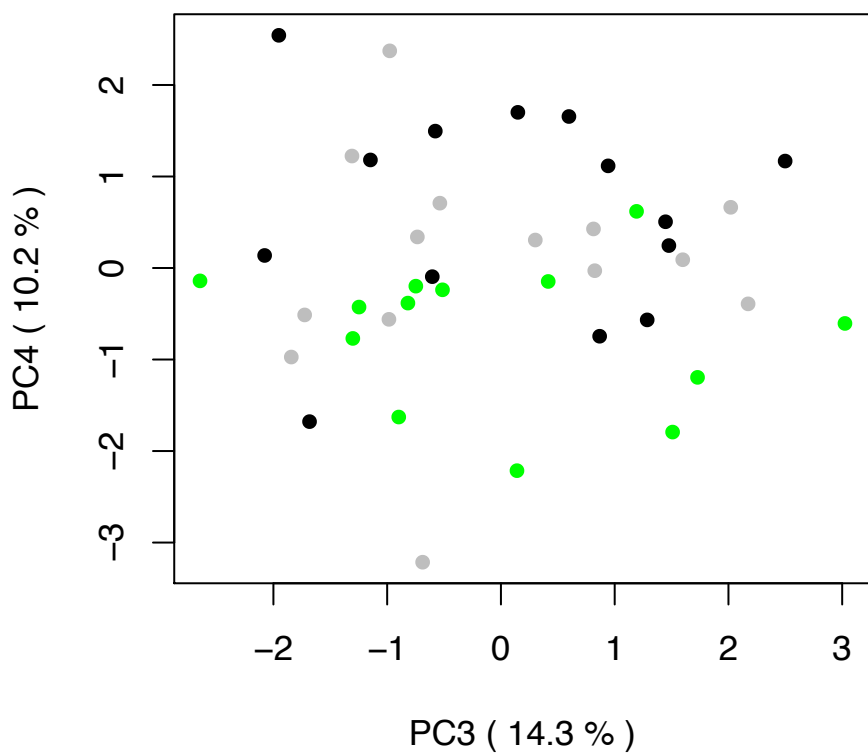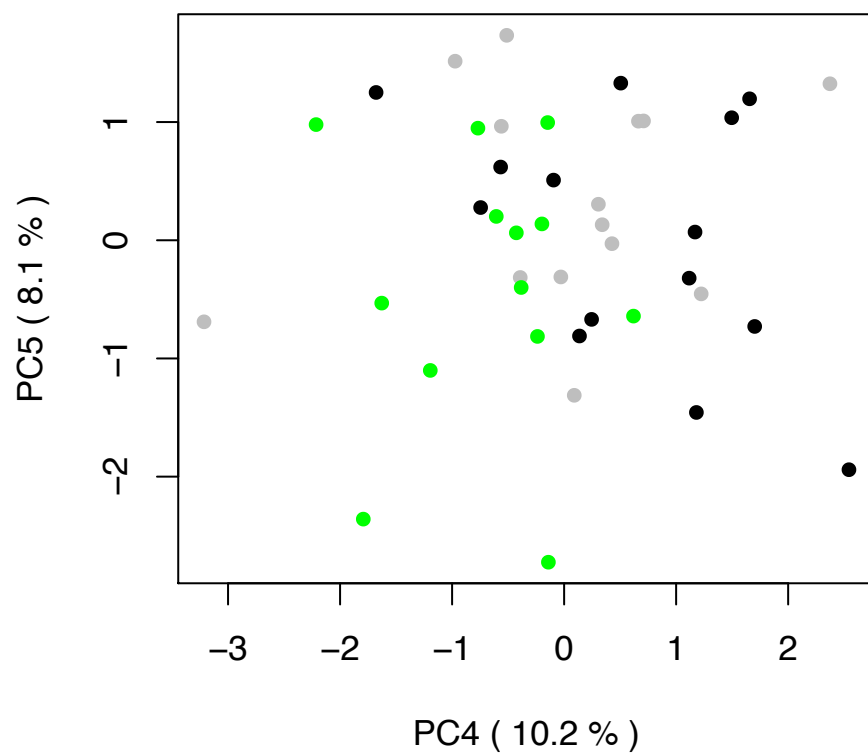

Supplement: S6 Fig — Enrichment data from 41 mice and 14 loci were used for PCA. Mice were assigned by body weight into three groups: black 20.0–22.4 g (n = 14), grey 22.5–24.9 g (n = 14), green 25.0–30.0 g (n = 13). (PDF) [file pone.0133988.s006.pdf]

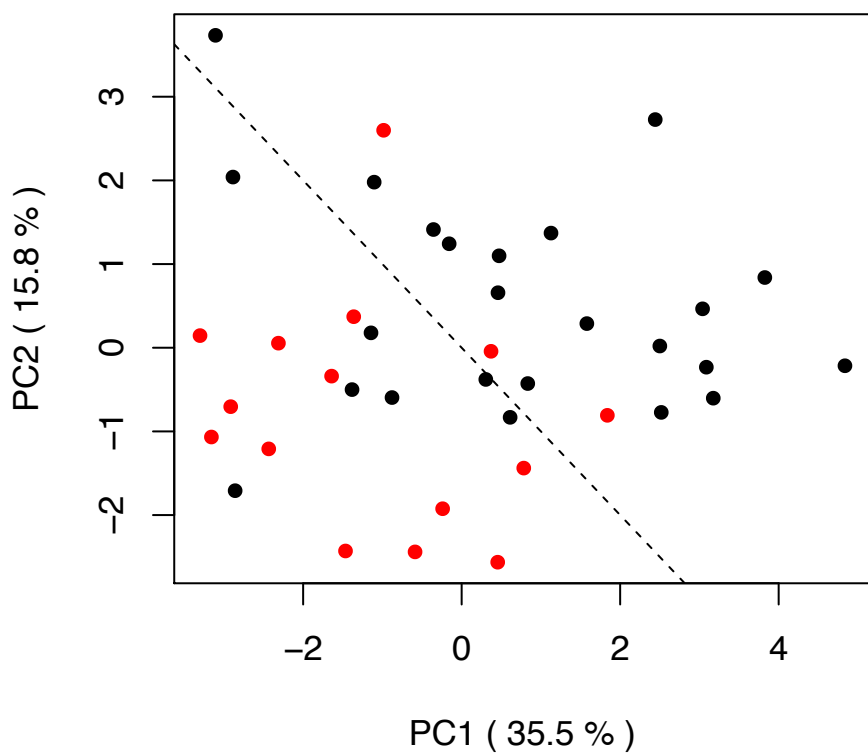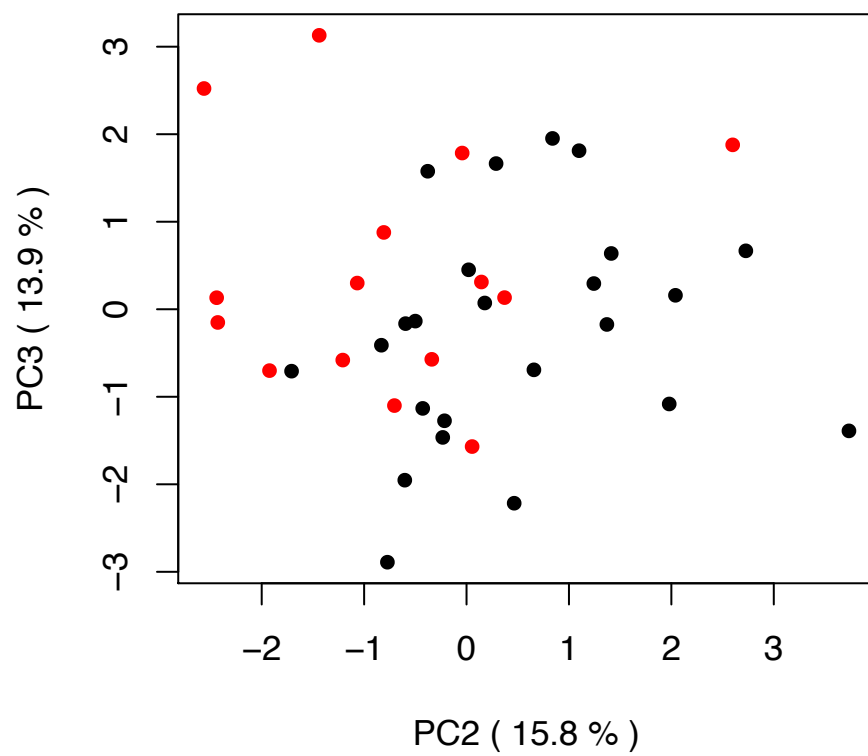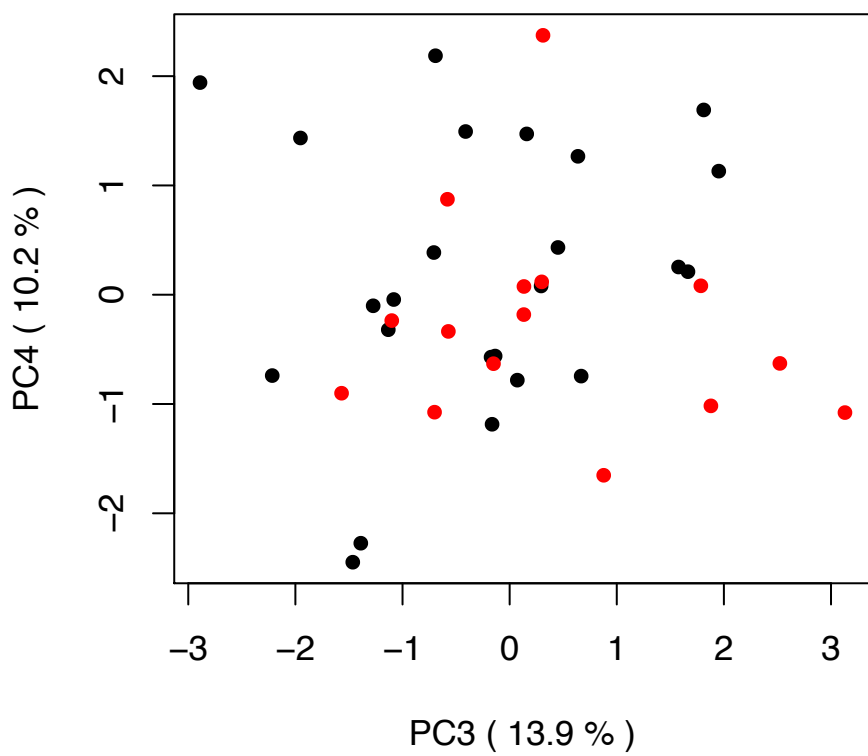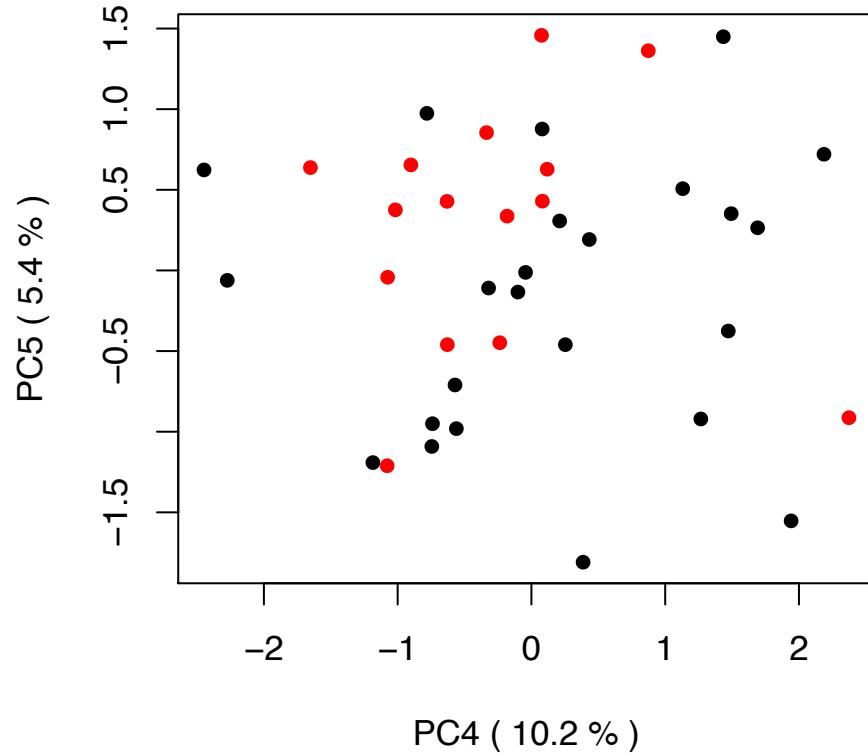

Supplement: S7 Fig — The dashed line is drawn through the origin of the plot PC1 vs. PC2 with a slope of -1 and shows the group separation that has been used for the permutation test. Three permutation runs of this separation by PC1 and PC2 gave a mean P-value of 3.1x10^-3^. When the dataset was divided into two groups at the level of PC1 = 0, this separation was marginally significant (p = 4.8x10^-2^), but grouping at the level of PC2 = 0 did not give a significant result (p = 9.8x10^-2^). (PDF) [file pone.0133988.s007.pdf]

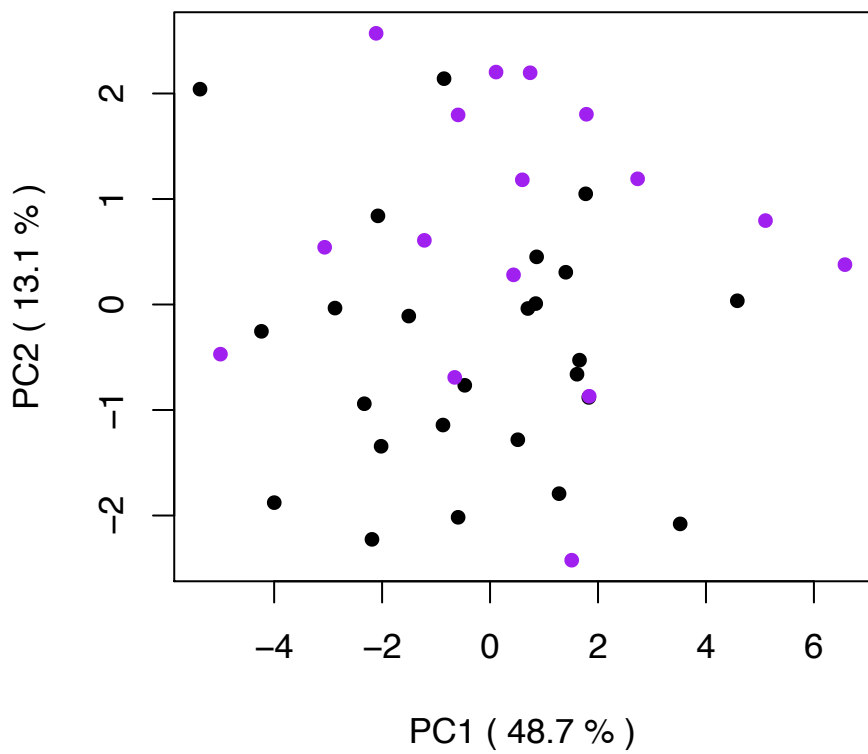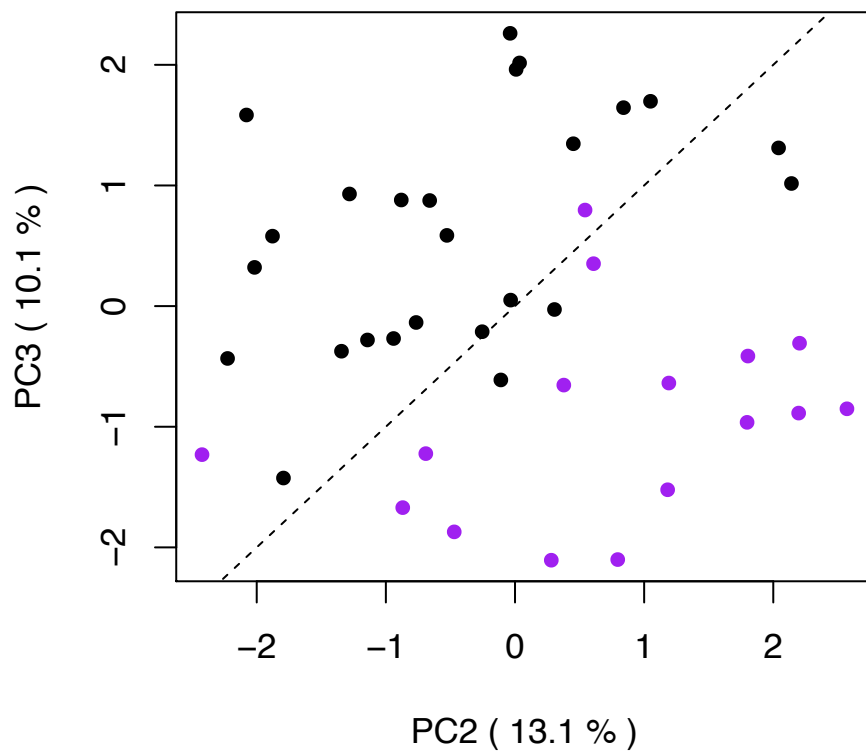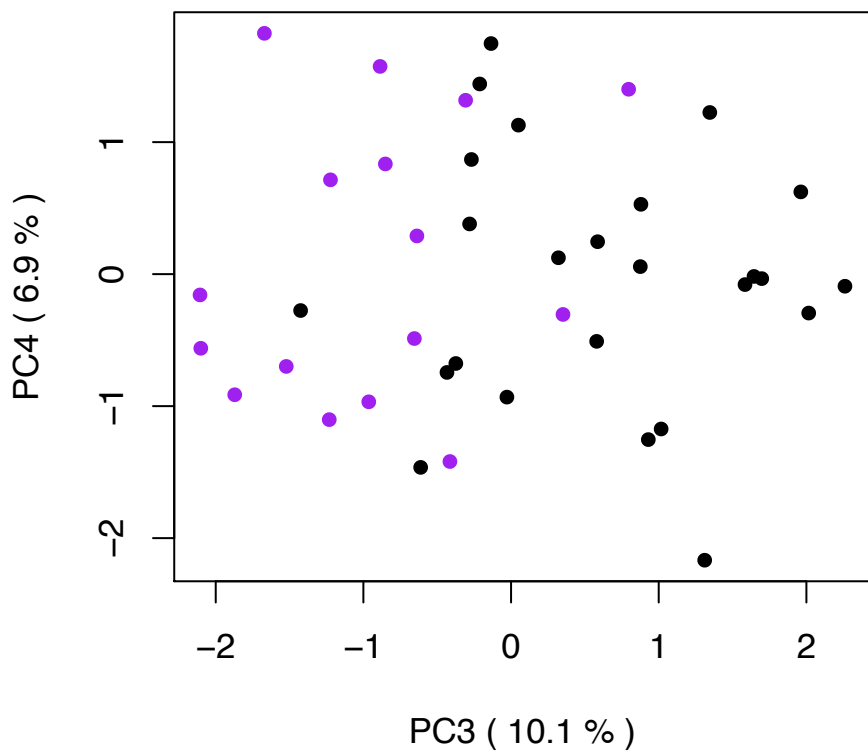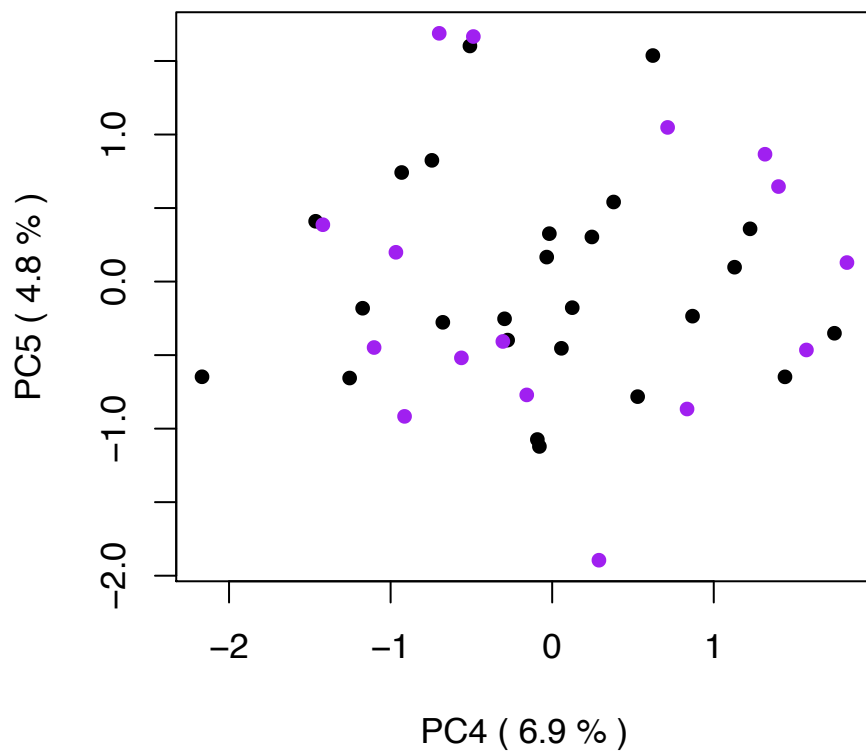

Supplement: S8 Fig — The dashed line is drawn through the origin of the plot PC2 vs. PC3 with a slope of +1 and shows the group separation that has been used for the permutation test. Three permutation runs of this separation by PC2 and PC3 gave a mean P-value of 1.1x10^-5^. When the dataset was divided into two groups at the level of PC2 = 0, the mean P-value was 2.2x10^-2^; when it was divided at the level of PC3 = 0, the mean P-value was 1.4x10^-3^. (PDF) [file pone.0133988.s008.pdf]

**A**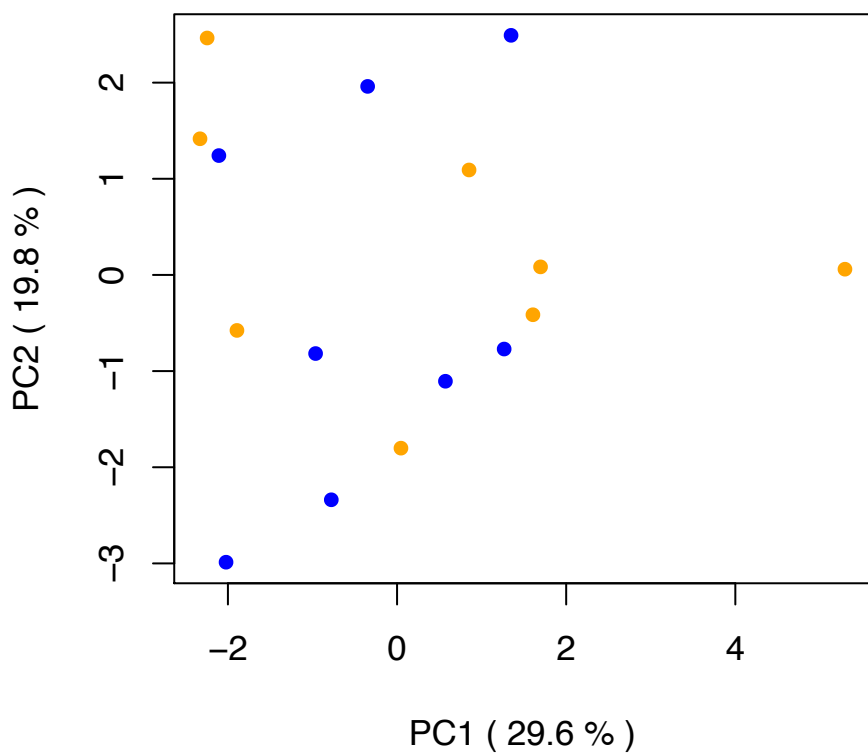**B**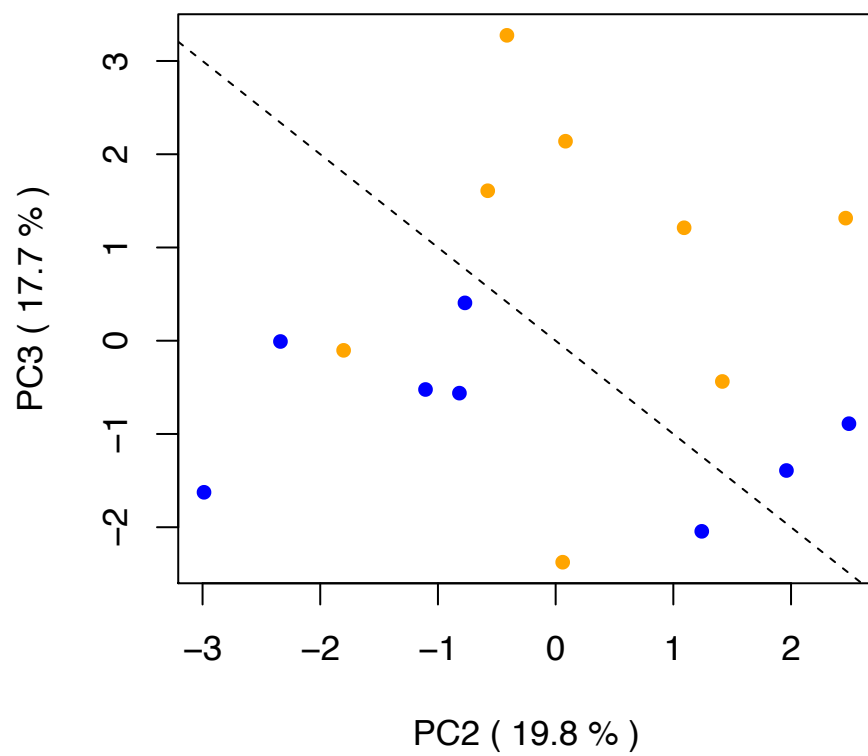**C**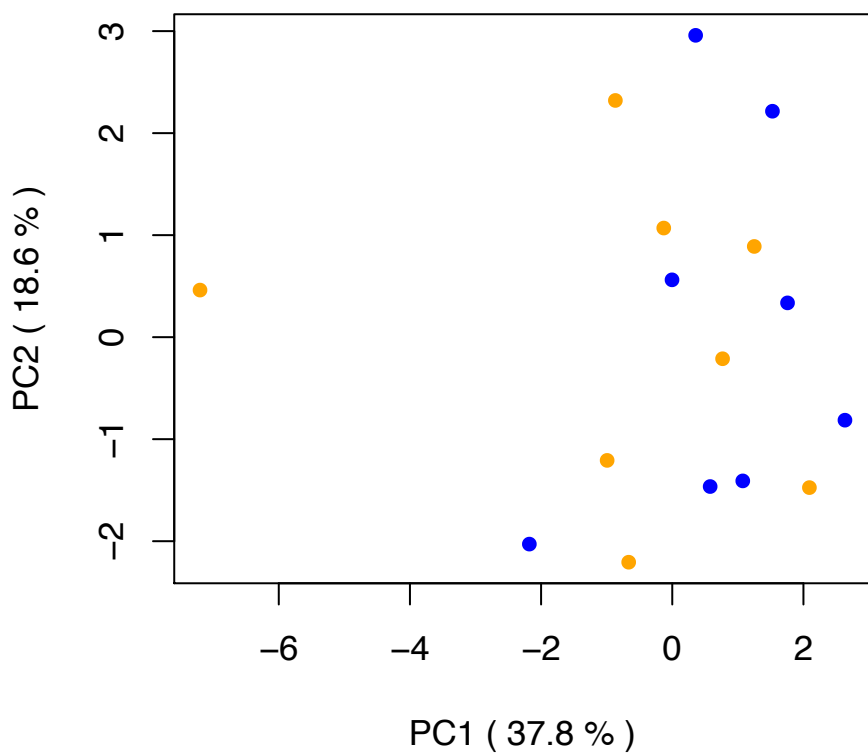**D**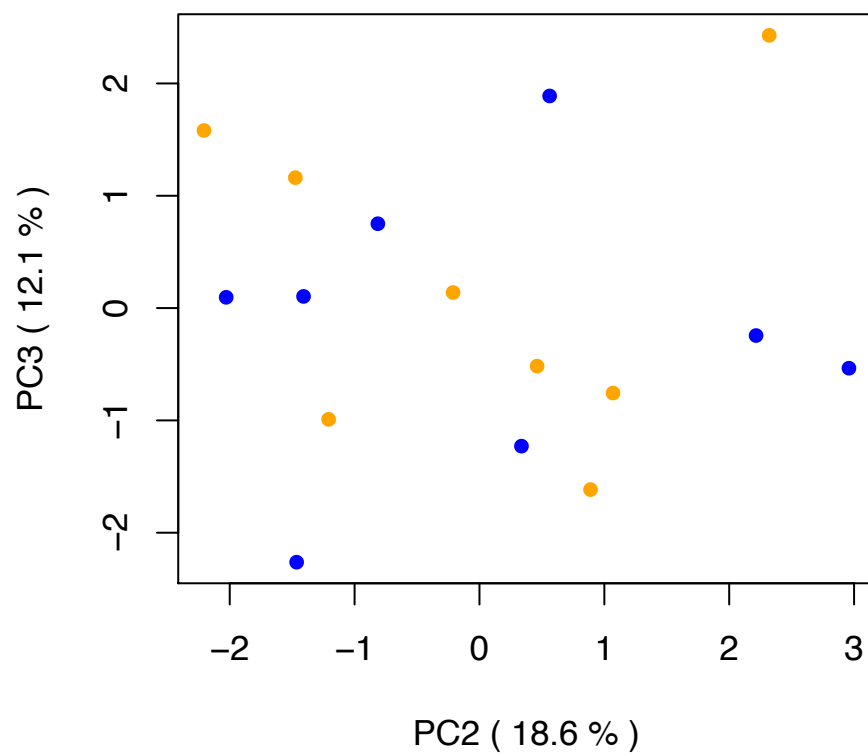

Supplement: S9 Fig — The percentage is the fraction of variance that is explained by the respective PC. Blue are mice from nest A, orange are mice from nest H. (A, B) H3K4me3 enrichment data, (C, D) H3K27ac enrichment data. (B) The dashed line shows a visual separation that splits the sample into two groups of 8 individuals each. This separation was tested by permutation and was found not to be significant (mean P-value of three runs: 0.13). (PDF) [file pone.0133988.s009.pdf]

**A**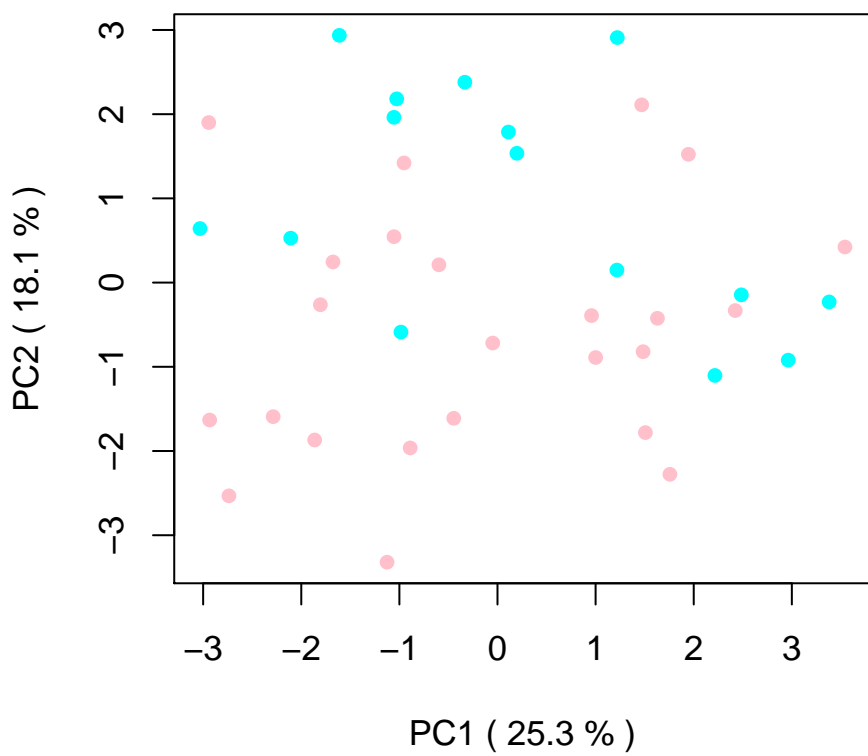**B**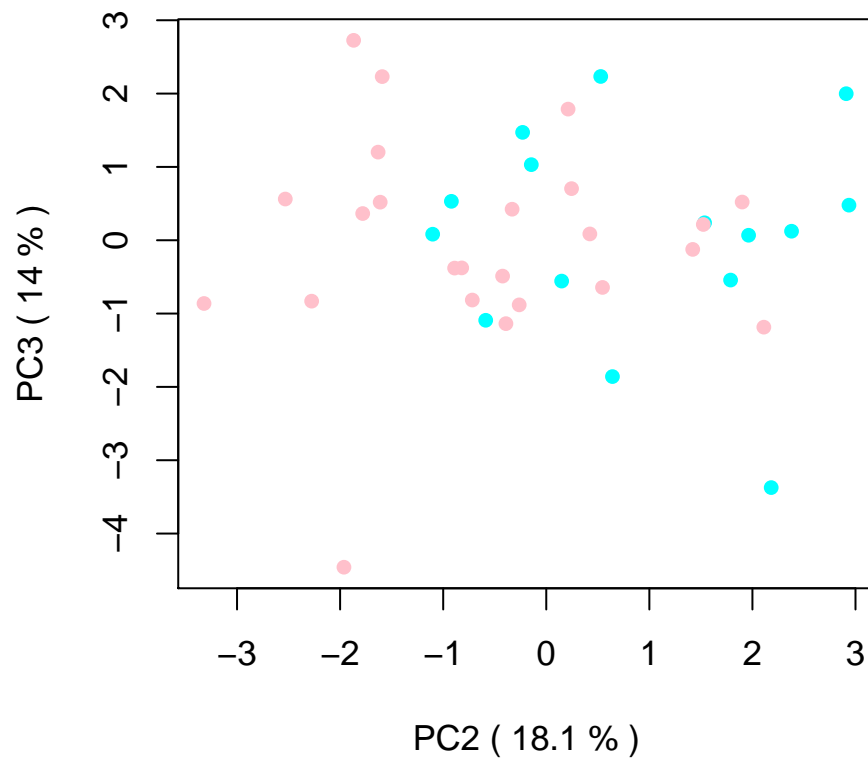**C**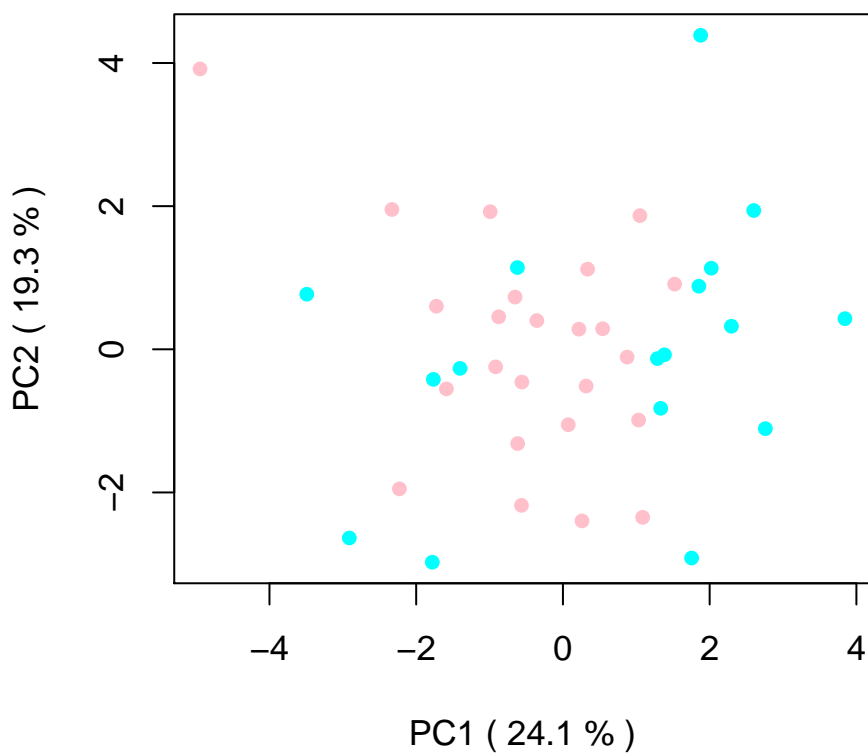**D**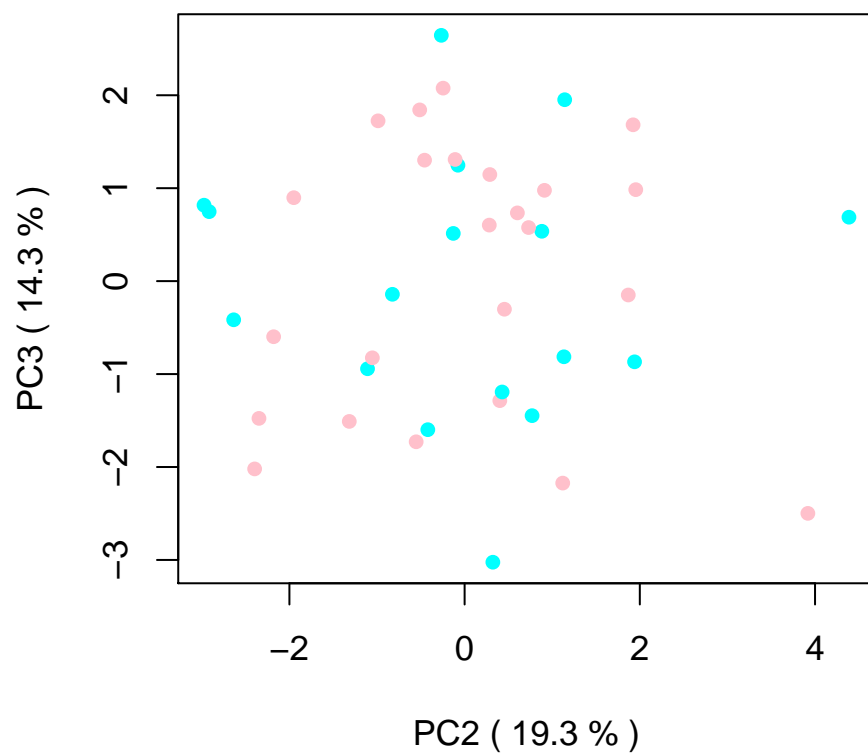

Supplement: S10 Fig — (A, B) H3K4me3 data of 39 mice were analyzed. (C, D) H3K27ac data of 41 mice were analyzed. Samples from preparation 1 are pink, samples from preparation 2 are cyan. (A) The significance of a possible separation of H3K4me3 data along PC2 = 0 was tested by permutation. The mean P-value of three runs was 5.5x10^-2^. (PDF) [file pone.0133988.s010.pdf]

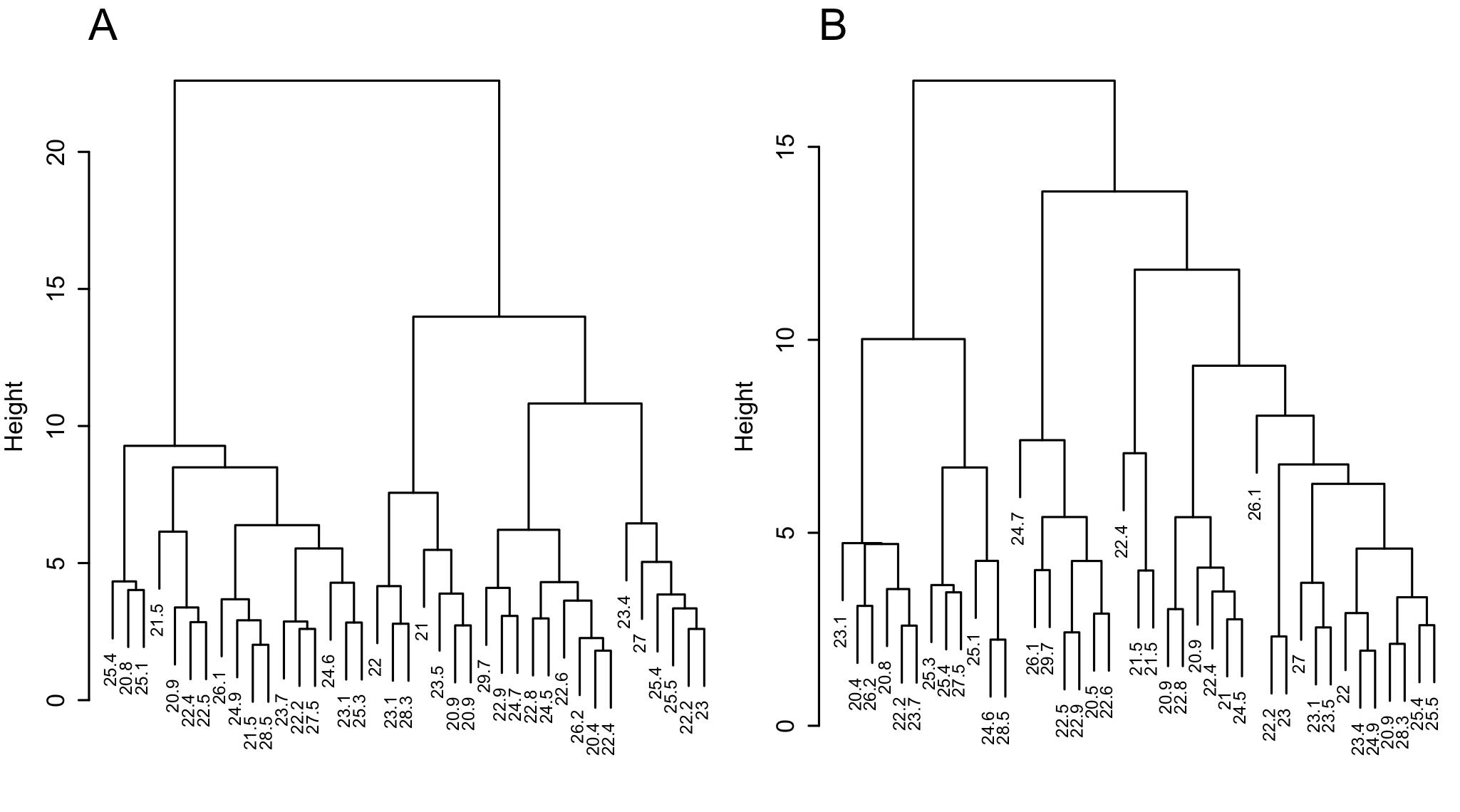

Supplement: S11 Fig — The analysis was performed in (A) from the H3K4me3 dataset with 39 mice (24 mice socialized, 15 mice ostracized) and in (B) from the H3K27ac dataset with 41 mice (25 mice socialized, 16 mice ostracized). Leaf labels are body weight in g. The mean body weights of the two largest clusters were 23.9 ± 2.3 and 23.7 ± 2.5 (mean ± sd in A) and 24.4 ± 2.5 and 23.5 ± 2.3 (in B). Means were not different as determined by a Welch two sample t-test and a permutation test. (TIF) [file pone.0133988.s011.tif]

**A**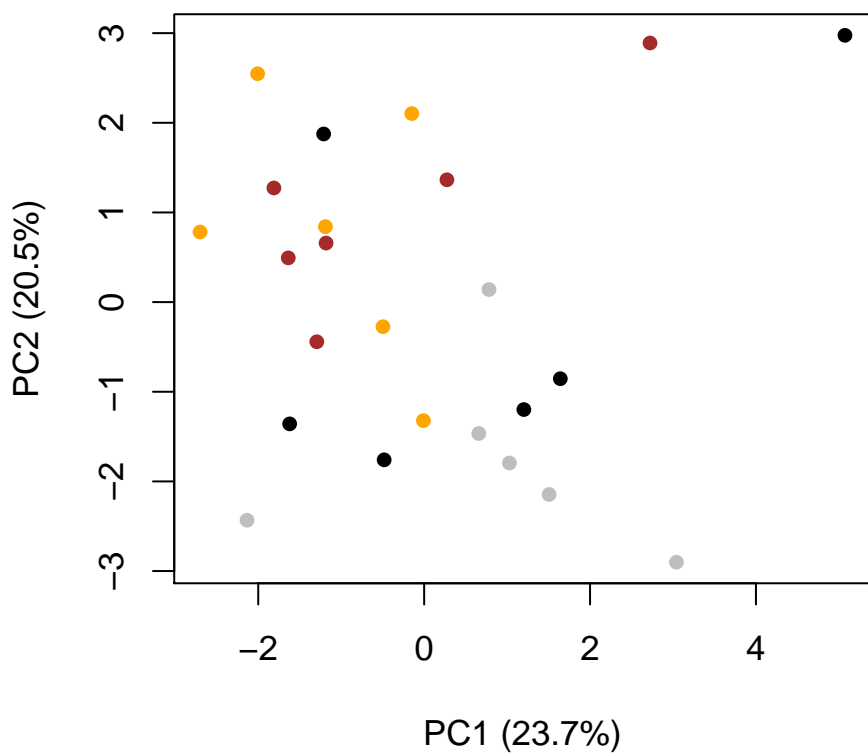**B**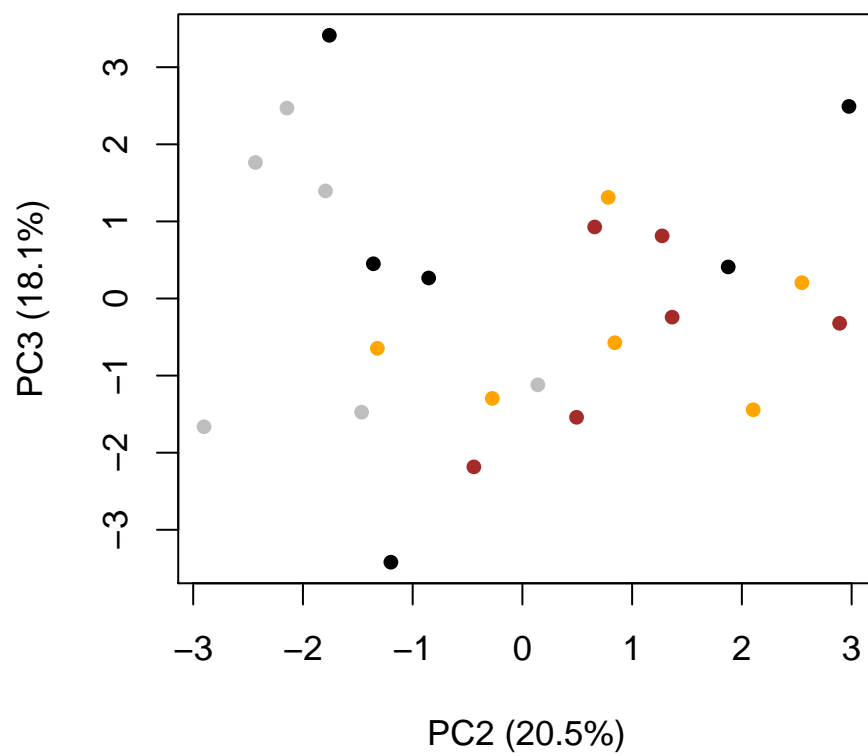**C**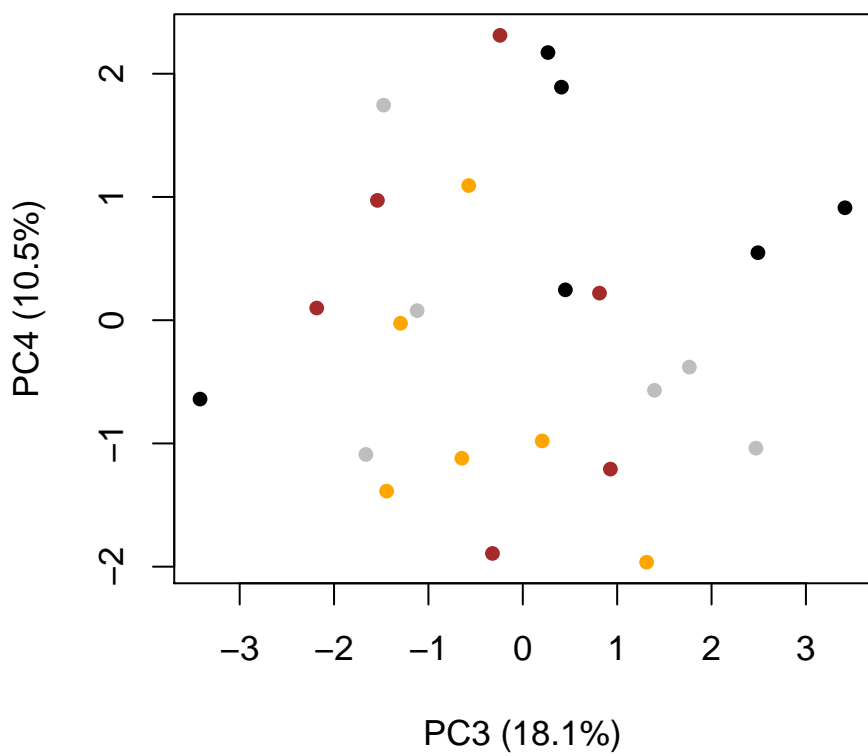**D**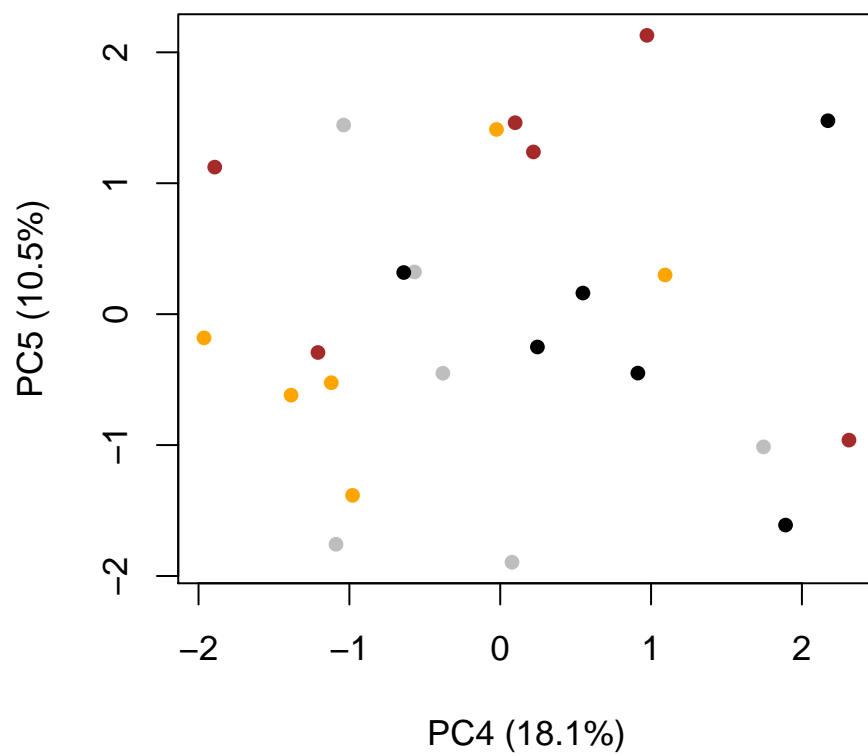

Supplement: S12 Fig — H3K4me3 qPCR enrichment data from 24 healthy females that lived in two different environments [10] were analyzed by PCA. Grey are young females living under standard conditions (body weight 17–20 g) and black are older females from the same environment (22–32 g). Orange are younger females living in a summer environment with an energy-enriched diet (body weight 16–18 g) and brown are older females from the same environment (21–30 g). The 15 gene loci of this dataset were Gapdh, Cdkn1a, Cyp2j5, Igf1, Igfbp2, Insig2, Plin5, Pop4, Ppara, Rpp21, Rsl1d1, Serpina6, Slc38a3, Smtn, and Tgfbr2. The two different environments had a stronger effect on group separation than the age of the animals. (PDF) [file pone.0133988.s012.pdf]

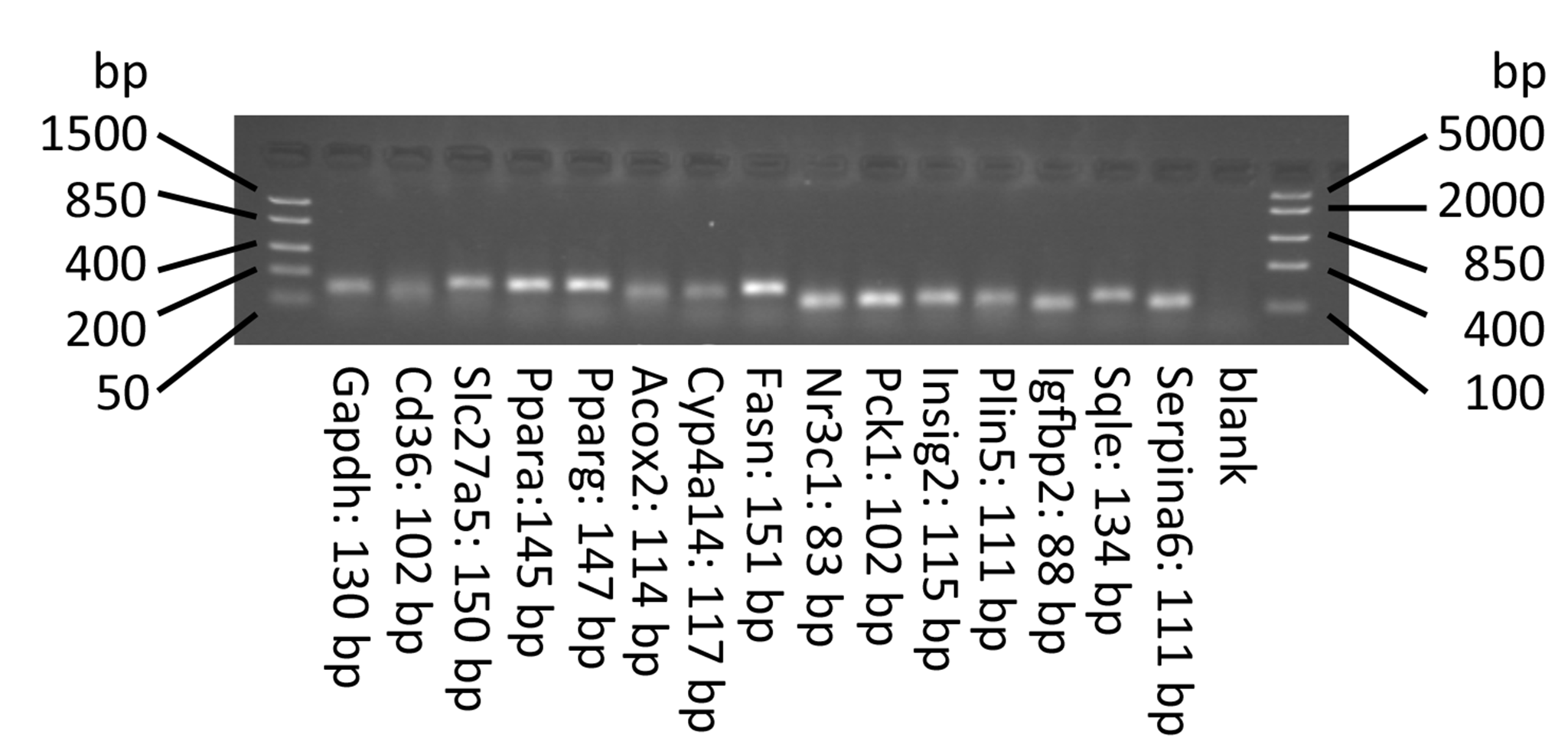

Supplement: S13 Fig — PCR products from one of the wild mouse chromatin preparations were separated on a 2% agarose gel and visualized using Sybr Green under UV. All PCR products were at the correct position according to the calculated amplicon lengths. (TIF) [file pone.0133988.s013.tif]

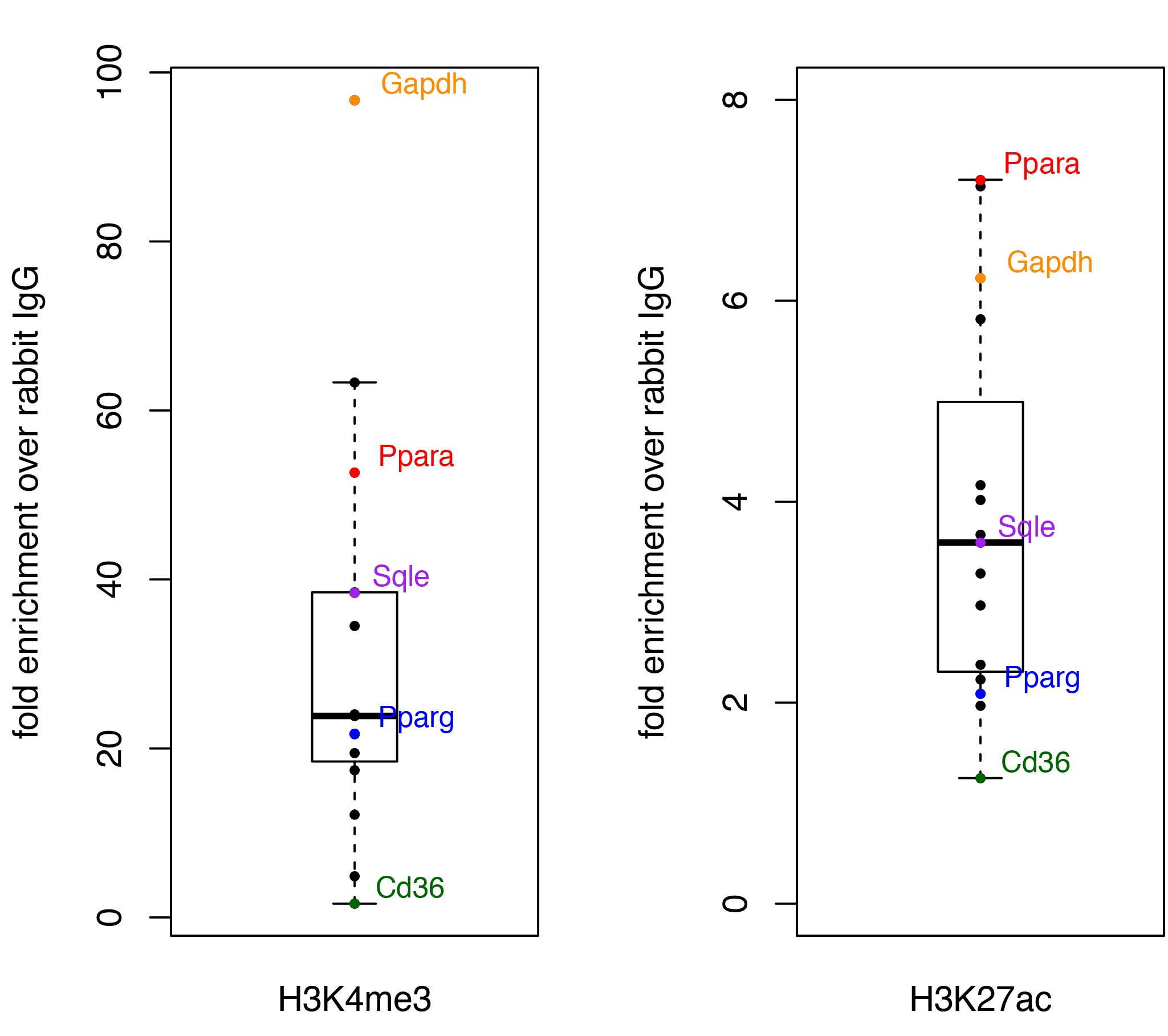

Supplement: S14 Fig — Boxplots show mean fold enrichment values of H3K4me3 and H3K27ac immunoprecipitates over rabbit IgG immunoprecipitates obtained from 41 samples. The ranking within the set of 15 loci is different between H3K4me3 and H3K27ac immunoprecipitates. (TIF) [file pone.0133988.s014.tif]
