# Supplementary material for: Social Exclusion Changes Histone Modifications H3K4me3 and H3K27ac in Liver Tissue of Wild House Mice
Source: PLoS One. 2015 Aug 12;10(8):e0133988. doi: 10.1371/journal.pone.0133988 (PMC4534140; doi:10.1371/journal.pone.0133988)
Supplement: S1 Table — (DOCX) [file pone.0133988.s015.docx]

**S1 Table. Body weights and fur condition of mice analysed in this study.**

| Preparation | Location^a^ | Mouse ID | Fur condition | Weight (g) | Mean±sd weight/  location (g) |
| --- | --- | --- | --- | --- | --- |
| 1 | A | a1 | good | 22.4 |  |
|  | A | a2 | good | 21.5 |  |
|  | A | a3 | good | 23.0 |  |
|  | A | a4 | good | 22.0 |  |
|  | A | a5 | good | 23.1 |  |
|  | A | a6 | good | 28.3 |  |
|  | A | a7 | good | 22.8 |  |
|  | A | a8 | good | 24.5 | 23.4 ± 2.1 |
|  | H | h1 | good | 20.9 |  |
|  | H | h2 | good | 22.2 |  |
|  | H | h3 | good | 22.4 |  |
|  | H | h4 | good | 20.9 |  |
|  | H | h5 | good | 22.5 |  |
|  | H | h6 | good | 21.0 |  |
|  | H | h7 | good | 23.4 |  |
|  | H | h8 | good | 23.5 | 22.1 ± 1.1 |
|  | Z | z1 | bite wounds | 25.1 |  |
|  | Z | z2 | bite wounds | 20.8 |  |
|  | Z | z3 | bite wounds | 23.1 |  |
|  | Z | z4 | bite wounds | 24.9 |  |
|  | Z | z5 | bite wounds | 25.4 |  |
|  | Z | z6 | bite wounds | 22.2 |  |
|  | Z | z7 | bite wounds | 23.7 |  |
|  | Z | z8 | bite wounds | 28.5 | 24.2 ± 2.4 |
| 2 | E | e1 | good | 24.7 |  |
|  | E | e2 | good | 25.5 |  |
|  | E | e3 | good | 22.9 |  |
|  | E | e4 | good | 25.4 |  |
|  | F | f1 | good | 21.5 |  |
|  | G | g1^b^ | good | 20.5 |  |
|  | G | g2 | good | 22.6 |  |
|  | G | g3 | good | 20.9 |  |
|  | G | g4 | good | 20.4 | 22.7 ± 2.1 |
|  | Z | z9 | bite wounds | 29.7 |  |
|  | Z | z10 | bite wounds | 26.1 |  |
|  | Z | z11 | bite wounds | 25.3 |  |
|  | Z | z12 | bite wounds | 27.5 |  |
|  | Z | z13 | bite wounds | 24.6 |  |
|  | Z | z14 | bite wounds | 27.0 |  |
|  | Z | z15 | bite wounds | 26.2 |  |
|  | Z | z16^b^ | bite wounds | 26.1 | 26.6 ± 1.6 |

^a^A, E, F, G, H are nest boxes, Z are free-roaming mice at the time point of sampling.

^b^Omitted from H3K4me3 data analyses unless stated otherwise.
