# Supplementary material for: Social Exclusion Changes Histone Modifications H3K4me3 and H3K27ac in Liver Tissue of Wild House Mice
Source: PLoS One. 2015 Aug 12;10(8):e0133988. doi: 10.1371/journal.pone.0133988 (PMC4534140; doi:10.1371/journal.pone.0133988)
Supplement: S2 Table — (DOCX) [file pone.0133988.s016.docx]

**S3 Table. qPCR data analysis: means and standard deviations of two normalization methods.**

| Marker | H3K4me3 | | | | H3K27ac | | | |
| --- | --- | --- | --- | --- | --- | --- | --- | --- |
| Phenotype | Socialized mice n=24 | | Ostracized mice n=15 | | Socialized mice n=25 | | Ostracized mice n=16 | |
| Gapdh-normalized dataset | mean | sd | mean | sd | mean | sd | mean | sd |
| *Gapdh* | 1.00 | 0.00 | 1.00 | 0.00 | 1.00 | 0.00 | 1.00 | 0.00 |
| *Cd36* | 0.10 | 0.02 | 0.10 | 0.02 | 1.09 | 0.33 | 1.42 | 0.35 |
| *Slc27a5* | 0.80 | 0.13 | 0.77 | 0.09 | 2.65 | 0.48 | 2.68 | 0.47 |
| *Ppara* | 0.63 | 0.10 | 0.69 | 0.09 | 1.30 | 0.31 | 1.37 | 0.35 |
| *Pparg* | 0.54 | 0.11 | 0.46 | 0.15 | 0.75 | 0.23 | 0.87 | 0.28 |
| *Acox2* | 0.44 | 0.10 | 0.40 | 0.09 | 2.64 | 0.62 | 3.05 | 0.76 |
| *Cyp4a14* | 0.42 | 0.23 | 0.11 | 0.10 | 1.10 | 0.42 | 0.96 | 0.38 |
| *Fasn* | 0.76 | 0.10 | 0.65 | 0.13 | 1.23 | 0.24 | 1.07 | 0.25 |
| *Nr3c1* | 0.80 | 0.14 | 0.85 | 0.11 | 1.28 | 0.15 | 1.54 | 0.29 |
| *Pck1* | 0.60 | 0.09 | 0.50 | 0.05 | 1.50 | 0.28 | 1.43 | 0.27 |
| *Insig2* | 0.89 | 0.18 | 0.82 | 0.15 | 2.18 | 0.39 | 2.45 | 0.61 |
| *Plin5* | 0.58 | 0.14 | 0.48 | 0.10 | 1.62 | 0.37 | 1.30 | 0.26 |
| *Igfbp2* | 0.58 | 0.08 | 0.58 | 0.10 | 0.81 | 0.20 | 0.91 | 0.21 |
| *Sqle* | 0.73 | 0.09 | 0.77 | 0.09 | 1.00 | 0.21 | 1.14 | 0.30 |
| *Serpina6* | 0.67 | 0.16 | 0.56 | 0.11 | 1.23 | 0.25 | 1.23 | 0.26 |
| Mean-normalized dataset | mean | sd | mean | sd | mean | sd | mean | sd |
| *Gapdh* | 1.59 | 0.18 | 1.72 | 0.14 | 0.72 | 0.12 | 0.69 | 0.12 |
| *Cd36* | 0.15 | 0.03 | 0.18 | 0.02 | 0.76 | 0.17 | 0.94 | 0.11 |
| *Slc27a5* | 1.26 | 0.13 | 1.32 | 0.12 | 1.86 | 0.22 | 1.80 | 0.23 |
| *Ppara* | 0.99 | 0.14 | 1.19 | 0.15 | 0.91 | 0.16 | 0.91 | 0.11 |
| *Pparg* | 0.85 | 0.12 | 0.78 | 0.20 | 0.52 | 0.13 | 0.57 | 0.15 |
| *Acox2* | 0.70 | 0.11 | 0.69 | 0.14 | 1.84 | 0.25 | 2.03 | 0.43 |
| *Cyp4a14* | 0.64 | 0.30 | 0.20 | 0.19 | 0.76 | 0.21 | 0.64 | 0.19 |
| *Fasn* | 1.20 | 0.13 | 1.11 | 0.18 | 0.87 | 0.19 | 0.72 | 0.14 |
| *Nr3c1* | 1.26 | 0.19 | 1.46 | 0.19 | 0.91 | 0.11 | 1.04 | 0.18 |
| *Pck1* | 0.94 | 0.11 | 0.86 | 0.10 | 1.06 | 0.14 | 0.96 | 0.14 |
| *Insig2* | 1.39 | 0.24 | 1.40 | 0.21 | 1.53 | 0.17 | 1.62 | 0.26 |
| *Plin5* | 0.91 | 0.20 | 0.82 | 0.14 | 1.13 | 0.16 | 0.87 | 0.13 |
| *Igfbp2* | 0.91 | 0.13 | 0.99 | 0.11 | 0.56 | 0.10 | 0.61 | 0.11 |
| *Sqle* | 1.16 | 0.16 | 1.32 | 0.11 | 0.71 | 0.14 | 0.76 | 0.13 |
| *Serpina6* | 1.05 | 0.20 | 0.97 | 0.19 | 0.87 | 0.14 | 0.83 | 0.12 |
