# Supplementary material for: Social Exclusion Changes Histone Modifications H3K4me3 and H3K27ac in Liver Tissue of Wild House Mice
Source: PLoS One. 2015 Aug 12;10(8):e0133988. doi: 10.1371/journal.pone.0133988 (PMC4534140; doi:10.1371/journal.pone.0133988)
Supplement: S3 Table — (DOCX) [file pone.0133988.s017.docx]

**S5 Table. Comparison of P-values from permutation and conventional statistical tests.**

|  | Phenotype: means | | | Weight: means | | | Phenotype: variances | Weight: variances |
| --- | --- | --- | --- | --- | --- | --- | --- | --- |
|  | Permutation | Anova | T-Test/Welch | Permutation | Anova | T-Test/Welch | Permutation | Permutation |
| H3K4me3 | raw P^a^ | raw P^a^ | raw P^a^ | raw P^a^ | raw P^a^ | raw P^a^ | adjusted P^b^ | adjusted P^b^ |
| *Gapdh* | 0.02337 | 0.02284 | 0.02284 | 0.08114 | 0.08096 | 0.08096 | 0.555 | 0.865 |
| *Cd36*^c^ | 0.00972 | 0.00922 | 0.00922 | 0.21397 | 0.2156 | 0.21563 | 0.555 | 0.952 |
| *Slc27a5* | 0.10003 | 0.1001 | 0.10010 | 0.00930 | 0.00882 | 0.00882 | 0.960 | 0.865 |
| *Ppara* | 0.00013 | 0.00016 | 0.00016 | 0.88260 | 0.8821 | 0.88214 | 0.960 | 0.865 |
| *Pparg* | 0.22410 | 0.2237 | 0.27865 | 0.82113 | 0.8209 | 0.82091 | 0.555 | 0.865 |
| *Acox2* | 0.86173 | 0.8615 | 0.86147 | 0.66600 | 0.6636 | 0.66364 | 0.835 | 0.865 |
| *Cyp4a14* | 0.00003 | 0.00001 | 0.00001 | 0.04647 | 0.04564 | 0.04564 | 0.555 | 0.865 |
| *Fasn* | 0.07711 | 0.07746 | 0.07746 | 0.54217 | 0.5393 | 0.53933 | 0.555 | 0.865 |
| *Nr3c1* | 0.00400 | 0.00368 | 0.00368 | 0.71400 | 0.7158 | 0.71578 | 0.960 | 0.952 |
| *Pck1* | 0.02084 | 0.02072 | 0.02072 | 0.96400 | 0.9641 | 0.96411 | 0.960 | 0.865 |
| *Insig2* | 0.88333 | 0.881 | 0.88104 | 0.98600 | 0.9854 | 0.98536 | 0.960 | 0.952 |
| *Plin5* | 0.12350 | 0.1236 | 0.12360 | 0.95397 | 0.9541 | 0.95415 | 0.555 | 0.952 |
| *Igfbp2* | 0.06349 | 0.06334 | 0.06334 | 0.50077 | 0.5019 | 0.50186 | 0.943 | 0.865 |
| *Sqle* | 0.00174 | 0.00215 | 0.00215 | 0.34897 | 0.3475 | 0.34745 | 0.835 | 0.952 |
| *Serpina6* | 0.21480 | 0.215 | 0.21498 | 0.76520 | 0.7656 | 0.76557 | 0.960 | 0.865 |
| H3K27ac | raw P^a^ | raw P^a^ | raw P^a^ | raw P^a^ | raw P^a^ | raw P^a^ | adjusted P^b^ | adjusted P^b^ |
| *Gapdh* | 0.42307 | 0.4212 | 0.42122 | 0.11487 | 0.1138 | 0.11384 | 0.872 | 0.842 |
| *Cd36*^c^ | 0.00025 | 0.00031 | 0.00030 | 0.22887 | 0.2272 | 0.22723 | 0.872 | 0.832 |
| *Slc27a5* | 0.41520 | 0.4146 | 0.41457 | 0.14120 | 0.1408 | 0.14084 | 0.872 | 0.832 |
| *Ppara* | 0.96740 | 0.9668 | 0.96681 | 0.07023 | 0.07234 | 0.07513 | 0.872 | 0.647 |
| *Pparg* | 0.26987 | 0.2671 | 0.26071 | 0.72643 | 0.7241 | 0.72407 | 0.872 | 0.832 |
| *Acox2* | 0.06959 | 0.07033 | 0.11101 | 0.57817 | 0.5735 | 0.57504 | 0.562 | 0.285 |
| *Cyp4a14* | 0.07221 | 0.07225 | 0.07225 | 0.08397 | 0.08386 | 0.08386 | 0.872 | 0.832 |
| *Fasn* | 0.00660 | 0.00735 | 0.00735 | 0.97103 | 0.9709 | 0.97086 | 0.872 | 0.832 |
| *Nr3c1* | 0.00358 | 0.00451 | 0.01270 | 0.19010 | 0.1872 | 0.18717 | 0.872 | 0.647 |
| *Pck1* | 0.03076 | 0.03074 | 0.03074 | 0.06745 | 0.06727 | 0.06727 | 0.963 | 0.832 |
| *Insig2* | 0.18040 | 0.1807 | 0.18071 | 0.76023 | 0.7612 | 0.76118 | 0.562 | 0.285 |
| *Plin5* | 0.000004 | 0.000004 | 0.000004 | 0.00016 | 0.00024 | 0.00024 | 0.872 | 0.832 |
| *Igfbp2* | 0.13970 | 0.1399 | 0.13988 | 0.75633 | 0.7569 | 0.75687 | 0.872 | 0.832 |
| *Sqle* | 0.22903 | 0.2296 | 0.22963 | 0.50363 | 0.5052 | 0.50523 | 0.872 | 0.832 |
| *Serpina6* | 0.38437 | 0.3832 | 0.38325 | 0.30050 | 0.2994 | 0.29945 | 0.872 | 0.832 |

Phenotype comparisons were carried out between 24 socialized and 15 ostracized males (H3K4me3) and between 25 socialized and 16 ostracized males (H3K27ac). Weight comparisons were carried out between 16 light and 16 heavy males. Permutation tests for equality of means or variances were performed with 10,000,000 iterations per run; given is the mean P-value from 3 runs.

^a^Raw P-values are given for the purpose of a numerical comparison of three test methods.

^b^P-values were adjusted for testing at multiple loci by the Benjamini-Hochberg method.

^c^*Cd36* was not marked.
