# Supplementary material for: Social Exclusion Changes Histone Modifications H3K4me3 and H3K27ac in Liver Tissue of Wild House Mice
Source: PLoS One. 2015 Aug 12;10(8):e0133988. doi: 10.1371/journal.pone.0133988 (PMC4534140; doi:10.1371/journal.pone.0133988)
Supplement: S4 Table — (DOCX) [file pone.0133988.s018.docx]

**S6 Table. Manova analyses of H3K4me3 and H3K27ac enrichment data from socialized and ostracized mice.**

|  | H3K4me3 enrichment (n=39) | | H3K27ac enrichment (n=41) | |
| --- | --- | --- | --- | --- |
| Categories for comparison | Number of samples | P^adj^ | Number of samples | P^adj^ |
| Socialized vs. ostracized | n=24, n=15 | 6.9 x 10^-4^ | n=25, n=16 | 2.2 x 10^-5^ |
| Body weight: light vs. heavy | n=16, n=16 | 0.52 | n=16, n= 16 | 0.23 |
| 20.0-22.4 g mice vs. 25.0-30.0 g mice | n=13, n=12 | 0.53 | n=14, n=13 | 0.23 |
| Nest A vs. nest H | n=8, n=8 | 0.52 | n=8, n=8 | 0.65 |
| Socialized preparation 1 vs. preparation 2^a^ | n=8, n=8 | 0.52 | n=8, n=9 | 0.65 |
| Ostracized preparation 1 vs. preparation 2 | n=8, n=8 | 0.53 | n=8, n=8 | 0.72 |
| Preparation 1 vs. preparation 2^b^ | n=16, n=15 | 0.21 | n=16, n=17 | 0.65 |

Manova tests were performed with data of 14 loci. P-values were adjusted by the Benjamini-Hochberg method to correct for multiple comparisons.

^a^Nest A vs. nests E/F/G.

^b^Nest H and ostracized from preparation 1 vs. nests E/F/G and ostracized from preparation 2.
