# Supplementary material for: Social Exclusion Changes Histone Modifications H3K4me3 and H3K27ac in Liver Tissue of Wild House Mice
Source: PLoS One. 2015 Aug 12;10(8):e0133988. doi: 10.1371/journal.pone.0133988 (PMC4534140; doi:10.1371/journal.pone.0133988)
Supplement: S5 Table — (DOCX) [file pone.0133988.s019.docx]

**S17 Table. Primer sequences for the qPCR analysis.**

| locus | forward primer sequence 5'-3' | reverse primer sequence 5'-3' | Tm_F (°C) | Tm_R (°C) | amplicon size | primer efficiency^a^ |
| --- | --- | --- | --- | --- | --- | --- |
| *Gapdh* | GCACCAGCATCCCTAGACC | GTGCAGTGCCAGGTGAAAAT | 60.64 | 61.11 | 103 | 2.045 |
| *Cd36* | ACTTGTGGCAAACAGGGCTGGAG | AGTCCGATGAGAGAGTGCAAGGCC | 59.56 | 60.29 | 102 | 2.183^b^ |
| *Slc27a5* | AGCCACATCTTTATGCAGCCAGCG | CTTGTGCTGCTTGGCTTGGCATTG | 60.12 | 60.00 | 150 | 2.413^b^ |
| *Ppara* | GTCTGGAGACCCACAGCCACT | AAACAGCTGCGAACACCAATGT | 63.98 | 64.08 | 145 | 1.982 |
| *Pparg* | GGCTTGTGGGGTCTGGATCTGACT | GAGTGTGGCTTTCCAGCCCGTATC | 59.94 | 59.59 | 147 | 1.988 |
| *Acox2* | CCTTTGTCTGCCTGCCTTCCTGTG | CTGACCAGGAGAGTTGGGGAGCAG | 59.94 | 60.23 | 114 | 2.431^b^ |
| *Cyp4a14* | TGCATGGGAAATGCTGGAGGGTCT | TCTCTGGGTTCTTCCAATGGGCCT | 60.17 | 59.32 | 117 | 2.075 |
| *Fasn* | ACACTTGACCTGGCCTGACCCTAC | AGTGCCCCAGACCCTGTTTCTTGA | 59.88 | 59.75 | 151 | 2.011 |
| *Nr3c1* | AGCCAGATAAACAAGTCGGCGTGC | TGGAAAAGAGGGGGCGACTGTTGA | 60.06 | 60.06 | 83 | 2.012 |
| *Pck1* | AGGTTCCCAGGGTGCATGAAAGGT | GCTTTGCAGCTCAGGTCGCCATTA | 60.05 | 60.06 | 102 | 2.006 |
| *Insig2* | CTGCGGAGGGCAGGCTGAAGAA | CCTCCCCTCTTCCCTCCACTCATCC | 61.07 | 61.04 | 115 | 2.019 |
| *Plin5* | GCAGGTCGCCTACCAAAGGCTGC | TGCAGGTCACCCAAACAGCCCCC | 62.40 | 63.28 | 111 | 2.023 |
| *Igfbp2* | CTGTGAGCACTAGTTTTGGGCTTG | CTCTCTTTTCCCACGTGAGAGTCA | 64.4 | 64.2 | 88 | 2.028 |
| *Sqle* | TGGTGACAAAAGACCGTTTACAGC | CAACGGCTCCTGATTACACACTTC | 64.5 | 64.2 | 134 | 2.009 |
| *Serpina6* | AAAAATCTCAAGCAAGCAGGCACT | AGACGTGAGTCACCCCTGACAGTA | 64.63 | 64.47 | 111 | 2.025 |

^a^Primer efficiencies are mean values from four tests on two inbred and two wild mouse chromatin preparations.

^b^Efficiencies calculated from the 2% and 20% input preparations only were 2.122 (*Cd36*), 2.096 (*Slc27a5*), and 2.177 (*Acox2*). Ct values of our measurements fell within the range of 2% and 20% input Ct values.
